# Regulatory roles of tankyrase 1 at telomeres and in DNA repair: suppression of T-SCE and stabilization of DNA-PKcs

**DOI:** 10.18632/aging.100210

**Published:** 2010-10-20

**Authors:** Ryan C. Dregalla, Junqing Zhou, Rupa R. Idate, Christine L.R. Battaglia, Howard L. Liber, Susan M. Bailey

**Affiliations:** Department of Environmental & Radiological Health Sciences, Colorado State University, Fort Collins, CO 80523-1618, USA

**Keywords:** tankyrase 1, DNA-PKcs, PARsylation, radiosensitivity, T-SCE, aging

## Abstract

Intrigued by the dynamics of the seemingly contradictory yet integrated cellular responses to the requisites of preserving telomere integrity while also efficiently repairing damaged DNA, we investigated roles of the telomere associated poly(adenosine diphosphate [ADP]-ribose) polymerase (PARP) tankyrase 1 in both telomere function and the DNA damage response following exposure to ionizing radiation. Tankyrase 1 siRNA knockdown in human cells significantly elevated recombination specifically within telomeres, a phenotype with the potential of accelerating cellular senescence. Additionally, depletion of tankyrase 1 resulted in concomitant and rapid reduction of the nonhomologous end-joining protein DNA-PKcs, while Ku86 and ATM protein levels remained unchanged; DNA-PKcs mRNA levels were also unaffected. We found that the requirement of tankyrase 1 for DNA-PKcs protein stability reflects the necessity of its PARP enzymatic activity. We also demonstrated that depletion of tankyrase 1 resulted in proteasome-mediated DNA-PKcs degradation, explaining the associated defective damage response observed; i.e., increased sensitivity to ionizing radiation-induced cell killing, mutagenesis, chromosome aberration and telomere fusion. We provide the first evidence for regulation of DNA-PKcs by tankyrase 1 PARP activity and taken together, identify roles of tankyrase 1 with implications not only for DNA repair and telomere biology, but also for cancer and aging.

## INTRODUCTION

Telomeres are specialized nucleoprotein structures composed of tandem arrays of repetitive, species-specific G-rich sequence that cap the ends of linear chromosomes. As such, they represent an important line of defense against end-to-end fusion and other untoward acts of recombination. In that sense, their ability to function properly can be viewed as diagnostic of genomic stability, or lack thereof. Accurate repair, e.g., the correct rejoining of double-strand breaks (DSB), is also imperative for maintaining genomic stability. Although appropriately dealing with each of these two types of DNA ends in and of themselves is absolutely essential, an intriguing interdependence of telomere function and DNA DSB processing has been emerging [1]. We and others have shown that mammalian telomeric end-capping function requires proteins more commonly associated with repair; e.g., the non-homologous end-joining (NHEJ) protein complex, DNA-dependent protein kinase (DNA-PK) [2,3]. Our continued characterization of uncapped (as opposed to shortened) dysfunctional telomeres in cells deficient for the catalytic subunit of DNA-PK (DNA-PKcs) provided the first indication that such uncapped telomeres are inappropriately detected and processed as DSBs [4], as well as evidence of their contribution to genomic instability and carcinogenesis, specifically murine mammary carcinoma following exposure to ionizing radiation (IR) [5]. Additional support for the role of altering proteins that protect chromosomal termini (genetic susceptibility) without shortening telomeres (uncapping) in accelerating tumorigenesis has recently been provided by the demonstration that mutation of the telomere protein TPP1 in mice resulted in carcinoma of the skin [6].

Utilizing Chromosome Orientation Fluorescence In Situ Hybridization (CO-FISH) [7], a strand-specific modification of standard FISH that provides information not available by any other means, we were the first to suggest differences in the post-replicative processing of leading- and lagging-strand telomeres [8], a view now supported by a variety of other studies [9,10]. In addition to its ability to distinguish between leading- vs. lagging-strand telomeres, CO-FISH also makes possible detection of sister chromatid exchange (SCE)-like recombination between telomeres, events termed T-SCE [11]. T-SCE are emerging as important features of telomerase negative backgrounds, such as alternative lengthening of telomeres (ALT) [12,13], early embryogenesis, prior to activation of telomerase [14], and pre-mature aging syndromes [15]. Interestingly, in the context of combined Wrn helicase deficiency and limiting telomere reserves (telomerase deficiency), both central features of Werner Syndrome pathogenesis, elevated telomere recombination (T-SCE) was observed, which was associated with greater immortalization potential [16]. It has also been shown that conditional deletion of the single-stranded telomere binding protein Pot1a elicits a DNA damage response at mouse telomeres, as well as aberrant homologous recombination manifested as increased T-SCE [17].

Early in our search for genes that regulate T-SCE frequencies, we examined the DNA repair protein poly(adenosine diphosphate [ADP]-ribose) polymerase1 (PARP1), as it was well established that genomic SCE (G-SCE) frequencies were greatly elevated in the context of PARP1 deficiency [18,19]. Treatment of PARP1-/- mouse cells with the PARP inhibitor 3-aminobenzamide (3-AB) elevated T-SCE, suggesting that PARP activity (other than PARP1) was normally and specifically suppressing T-SCE frequencies [11]. Tankyrase 1 provided an attractive candidate, as it had been shown to be a telomere associated PARP that complexes with TRF1 [20,21]. Tankyrase 1 regulates the amount of TRF1 at the telomere via poly(ADP-ribosyl)ation (PARsylation), a modification that releases TRF1 from the telomere, thereby controlling access and elongation by telomerase [22]. More recently, tankyrase 1 has been shown to function in sister telomere separation/resolution, as depletion of tankyrase 1 in human cells resulted in sister telomeres “cohering” at mitosis [23]. Knockout mice deficient in tankyrase 1, and the closely related tankyrase 2, have also been generated and revealed that the tankyrases are essential but redundant for murine development, as the single mutants display no telomere phenotype [24,25]. Consistent with this view, T-SCE frequencies were not elevated in tankyrase 2 mouse knockout cells (unpublished observation).

We hypothesized that there are likely to be functions of tankyrase 1 at human telomeres, and possibly elsewhere, which are independent of telomere length maintenance. To explore this possibility, we employed siRNA knockdown of tankyrase 1 in human cells, then examined relevant telomere and damage response end-points. We found that depletion of tankyrase 1 in telomerase negative backgrounds significantly elevated T-SCE levels, while G-SCE levels remained unchanged, demonstrating that tankyrase 1 normally suppresses recombination specifically within telomeric DNA. We also found that reduced levels of tankyrase 1 resulted in increased sensitivity to IR-induced cell killing, mutagenesis, chromosome aberration (terminal deletion), and telomere fusion, all suggestive of a role for tankyrase 1 in DNA repair. However, upon wondering whether NHEJ could be impaired, we discovered that depletion of tankyrase 1 resulted in rapid proteasome-mediated degradation of DNA-PKcs protein, while Ku86 and ATM protein levels remained unchanged, indicating that tankyrase 1 is required for DNA-PKcs protein stability. Further, DNA-PKcs mRNA levels were unaltered upon tankyrase 1 knockdown, revealing that DNA-PKcs regulation is not mediated through mRNA synthesis or stability, but rather via post-translational modification. Conclusive mechanistic insight was provided utilizing the tankyrase-specific PARP small molecule inhibitor XAV939 [26], which demonstrated similarly rapid and concomitant depletion DNA-PKcs protein levels as tankyrase 1 knockdown. Interestingly, tankyrase 1 protein levels increased with inhibition of its PARP activity, reflecting loss of tankyrase autoPARsylation, ubiquitnation and subsequent proteasome-mediated degradation [27], as well as supporting the specificity of XAV939 for the tankyrase PARP domain. Our results demonstrate that DNA-PKcs protein stability is specifically dependent on the PARsylation activity of tankyrase 1.

Previous studies have shown that covalent modification of DNA-PKcs via PARsylation stimulates its kinase activity in vitro [28], providing evidence of a functional role for PARslyated DNA-PKcs. Furthermore, DNA-PKcs has been identified as a member of the proteome associated with poly(ADP-ribose) protein complexes, indicating that DNA-PKcs exists in a PARsylated state in vivo [29]. Our findings are also consistent with reports demonstrating coordinate regulation of the phosphatidylinositol 3-kinase related kinases (PIKK) kinase family members (includes ATM, ATR and DNA-PKcs) by mammalian Tel2, which suggest specific dependence for protein stability [30,31]. Interestingly, it was recently reported that tankyrase 1 (and 2) interact with axin (Wnt signaling pathway), and in so doing, stimulate axin degradation via PARsylation and subsequent ubiquitination [26]. It has also been shown that stabilization of the nuclear mitotic apparatus protein (NuMa) is dependent on tankyrase 1 PARsylation [32]. Here, we provide the first evidence of tankyrase 1-PARP dependent stabilization of the NHEJ protein DNA-PKcs, revealing a new aspect of DNA-PKcs regulation that impacts both telomere and DNA repair functions.

## RESULTS

### Depletion of tankyrase 1 increases telomeric recombination (T-SCE)

Effective tankyrase 1 knockdown was consistently achieved one and two days after transfection with several different siRNAs, in multiple cell lines of various telomerase statuses. Two independent siRNAs reduced tankyrase 1 protein levels to <1 % of that observed in mock-transfected controls on numerous occasions (Figure S1). Tankyrase 1 siRNA 1 was then selected as the preferred reagent for the majority of the experiments described below.

**Figure 1.**
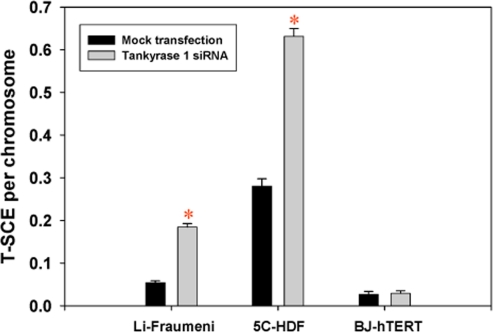
T-SCE frequencies are significantly elevated upon depletion of tankyrase 1 in telomerase negative backgrounds. T-SCE/chromosome levels were determined in three human fibroblast cell lines with various telomerase statuses; Li-Fraumeni 087 (ALT), 5C-normal human dermal (telomerase negative) and BJ-hTERT (5ta; telomerase positive). (*) is p<0.05.

T-SCE frequencies (T-SCE/chromosome) were evaluated following siRNA depletion of tankyrase 1 and were found to be consistently and significantly elevated in telomerase negative backgrounds (5C normal human dermal fibroblasts and Li Fraumeni 087, ALT) [11,33], but not in BJ-5ta telomerase positive cells (Figure 1). The finding of elevated T-SCE frequencies in telomerase negative backgrounds is consistent with our other studies investigating WRN, BLM and ERCC1 deficiencies [16,34]. G-SCE frequencies were also evaluated and remained unchanged with tankyrase 1 knockdown (data not shown). Together, these results demonstrate that tankyrase 1 normally acts to suppress SCE-like recombination specifically within telomeric DNA, and are especially interesting when contrasted with the established role of PARP1 in suppressing G-SCE [18,19], but not T-SCE [11].

### Depletion of tankyrase 1 increases sensitivity to IR-induced cell killing, gene mutation and chromosome aberration

Reduced survival, as determined by clonogenic assays, was observed following tankyrase 1 knockdown in human cells (Li Fraumeni 087 and 5C fibroblasts) and exposure to various doses of gamma (γ)-rays (Figure S2). Flow cytometric analyses confirmed cell cycle distributions were unaffected by tankyrase 1 knockdown, for both unirradiated and irradiated cells.

**Figure 2.**
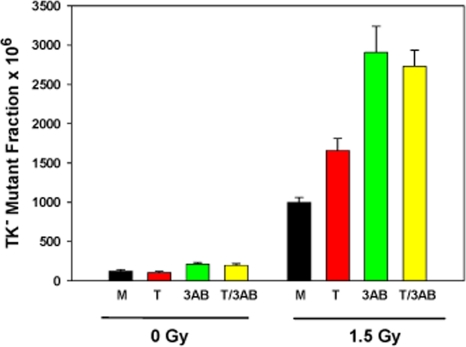
Increased γ-ray mutagenicity in WTK1 lymphoblasts upon tankyrase 1 siRNA depletion and/or inhibition of PARP with 3-AB. Cells were treated (representative knockdown, Figure S3), then irradiated the next day. Data are the average of three independent determinations; error bars are standard deviations. (M) mock transfection, (T) tankyrase 1 siRNA, (3-AB) inhibitor, and (T/3-AB) two combined.

Mutagenesis experiments were performed at the heterozygous thymidine kinase (TK) locus in WTK1 lymphoblasts after treatments with tankyrase 1 siRNA, the PARP inhibitor 3-AB, or the two combined (Figure 2). Cells were exposed to 0 or 1.5 Gy γ-rays 18 hours after treatment and the mutant fractions (MF) determined three days later. Background MFs were not affected by tankyrase 1 knockdown in these experiments (p=0.24), but inhibition of PARP activity with 3-AB resulted in a significant increase (p=0.004). Radiation-induced MFs were modestly elevated (1.5 times) after depletion of tankyrase 1 (p=0.002), but were even more so (2.5 times) with PARP inhibition (p<0.001). The combination of tankyrase 1 siRNA knockdown and PARP inhibition was equally effective as PARP inhibition alone (p=0.48). These results suggest the importance of PARP activity for reducing IR-induced MF and support the interpretation that tankyrase 1-mediated PARsylation is a key factor.

**Figure 3.**
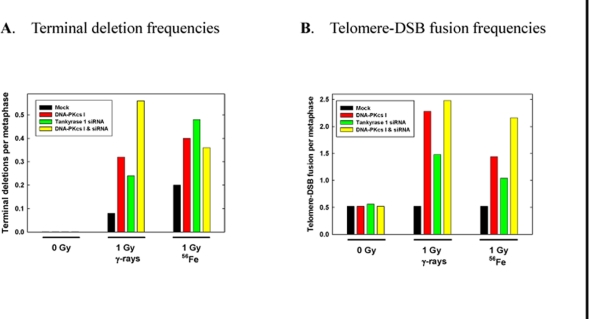
IR-induced chromosomal terminal deletions and telomere-DSB fusions are increased with tankyrase 1 siRNA knockdown. WTK1 lymphoblasts were treated on successive days with tankyrase 1 siRNA, or with the DNA-PKcs inhibitor (I), or with both, and irradiated (γ-rays or 1GeV ^56^Fe) 48 hr after the second transfection. (**A**) Frequencies of terminal deletion, hallmarks of defective NHEJ, following IR exposure were elevated with either DNA-PKcs inhibition or depletion of tankyrase 1. (**B**) Frequencies of IR-induced telomere-DSB fusions, events characteristic of telomere uncapping, were elevated with inhibition of DNA-PKcs, and also with depletion of tankyrase 1. These data represent single experiments.

Chromosome aberration assessment revealed significantly increased frequencies of IR-induced terminal deletions following tankyrase 1 siRNA knockdown and exposure to 1 Gy of either γ-rays or 1 GeV/n 56Fe ions (p < 0.03) (Figure 3). This aberration type reflects defective DSB repair; i.e., an inability to rejoin broken DNA ends. Interestingly, terminal deletion frequencies upon depletion of tankyrase 1 were not significantly different than those observed with inhibition of DNA-PKcs kinase activity (p>0.76), or the two treatments combined (p>0.18).

### Depletion of tankyrase 1 results in telomere uncapping

A significant increase in spontaneous telomere-telomere fusion with tankyrase 1 depletion was not observed, however, a telomeric uncapping phenotype was revealed following IR exposure; i.e., telomere-DSB fusion [4,5] (Figure 3). A three fold increase in telomere-DSB fusion frequency with γ-rays (p=0.023) and a two fold increase with 1 GeV ^56^Fe ions (p=0.14) was observed with tankyrase 1 depletion, increases similar to those seen with DNA-PKcs inhibition or the two treatments combined (all p < 0.023). Telomere-DSB fusion is consistent with the finding of increased terminal deletions, and is suggestive of problems not only with telomere end-capping, but with NHEJ DSB repair as well [1]. In fact, the instability phenotypes observed paralleled classic hallmarks of DNA-PKcs repair deficiency.

### Depletion of tankyrase 1 rapidly reduces DNA-PKcs protein levels, but does not affect Ku86 protein levels or DNA-PKcs mRNA levels

**Figure 4.**
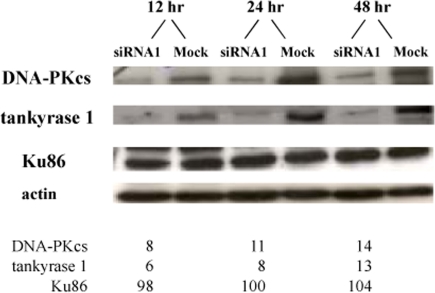
Tankyrase 1 depletion rapidly *reduces* DNA-PKcs protein levels, while Ku86 levels remain unchanged. Li-Fraumeni fibroblasts were transfected with tankyrase 1 siRNA or were mock transfected. Protein levels of DNA-PKcs, tankyrase 1, Ku86 and β-actin were determined by Western blot 12, 24 or 48 hr after transfection. Percentages of protein remaining are shown below; all values were normalized to β-actin and the mock transfection.

Western blot analyses of several human fibroblast and lymphoblast cell lines at various times (12, 18, 20, 24, and 48 hr) following transfection with two different tankyrase 1 siRNAs, revealed rapidly reduced levels of DNA-PKcs protein (exemplified in Figures 4 and S3), while protein levels of Ku86 and β-actin remained unchanged (Figure 4). Quantification of relative protein levels, normalized to the respective mock transfections and actin, confirmed this unexpected result. Monitoring the time-course of tankyrase 1 knockdown demonstrated not only rapid and concomitant loss of both tankyrase 1 and DNA-PKcs proteins (observed at 12 hr, as compared to 72 hr for DNA-PKcs knockdown; Figure S5), but also that as the levels of tankyrase 1 protein recovered (by ~72 hr), DNA-PKcs protein levels quickly rebounded (Figure S3), further supporting a direct connection between the two.

**Figure 5.**
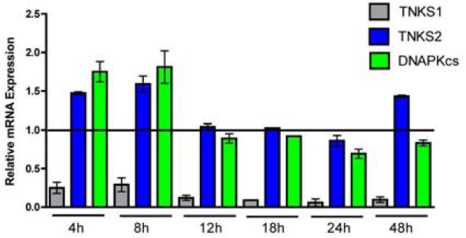
Time course of tankyrase 1 (TNKS1), tankyrase 2 (TNKS2) and DNA-PKcs relative mRNA expression following tankyrase 1 siRNA depletion. Quantitative RT-PCR of mRNA at 4, 8, 12, 18, 24 and 48 hr demonstrates dramatic reduction of tankyrase 1 mRNA (confirming efficiency of knockdown), as well as no significant reduction of tankyrase 2 (confirming specificity of knockdown) or DNA-PKcs (all p < 0.05).

We next asked whether mRNA levels were affected by tankyrase 1 siRNA knockdown. Determination of relative mRNA levels by quantitative Real-Time PCR (qRT-PCR) at various times post tankyrase 1 siRNA transfection (4, 8, 12, 18, 24 and 48 hr) confirmed, as expected, rapid and dramatic depletion of tankyrase 1 mRNA (Figure 5). We also established that there was no significant reduction of the closely related tankyrase 2 mRNA (all p > 0.05), supporting the specificity of tankyrase 1 siRNA knockdown. Likewise, there was no significant reduction of DNA-PKcs mRNA levels, signifying that the associated depletion of DNA-PKcs protein that occurs with loss of tankyrase 1 is not mediated by reduction of DNA-PKcs mRNA. Further, these results provide evidence that the observed instability phenotypes are the result of tankyrase 1 depletion.

### Tankyrase 1 stabilizes DNA-PKcs by protecting it from proteolytic degradation

**Figure 6.**
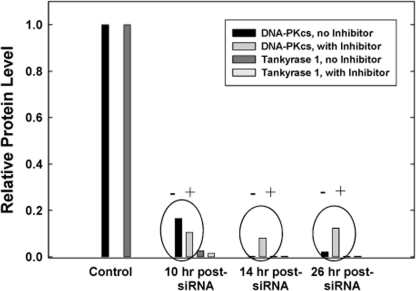
Proteasome inhibition facilitates DNA-PKcs protein recovery. Following siRNA depletion of tankyrase 1, WTK1 cells were treated (+) with the proteasome inhibitor MG132 for 2 hr at various times. Protein levels were measured at 10, 14 and 26 hr post transfection. When compared to untreated samples (-), protein levels of DNA-PKcs began to recover, suggesting tankyrase 1 prevents DNA-PKcs proteolytic degradation. Protein levels of tankyrase 1 were unaffected. Similar results were observed with the tankyrase PARP inhibitor XAV939 and MG132 treatment (Figure S4).

At various times post tankyrase 1 siRNA transfection (8, 12, and 24 hr), cells were treated with the proteasome inhibitor MG132 for two hour time intervals. As before, tankyrase 1 and DNA-PKcs protein levels plummeted. However, the two hour MG132 treatments resulted in recovery of DNA-PKcs protein to ~10-15% of the steady-state level, while tankyrase 1 protein levels were not affected and remained low (Figure 6). Similar results were also observed following treatment with the tankyrase specific PARP inhibitor XAV939 (12 hr) to reduce DNA-PKcs levels; i.e., DNA-PKcs protein levels recovered during 2 hr time intervals (Figure S4). These results demonstrate that inhibition of proteasome-mediated protein degradation allows cells to accumulate DNA-PKcs protein, and so provide support for the notion that tankyrase 1 protects DNA-PKcs from proteolytic degradation. This observation is also consistent with our qRT-PCR results demonstrating sufficient levels of DNA-PKcs mRNA following tankyrase 1 knockdown (Figure 5); i.e., ample DNA-PKcs message is available for translation. That DNA-PKcs protein levels were perhaps only minimally restored upon proteasome inhibition may reflect the short time allowed for recovery, that MG132 does not completely inhibit the proteasome, and/or that it takes time to synthesize such a large and abundant protein.

### Depletion of DNA-PKcs does not influence tankyrase 1 protein levels

To further investigate underlying mechanisms of the tankyrase 1 effect on DNA-PKcs stability, we performed the converse experiment; i.e. DNA-PKcs siRNA knockdown and monitoring of protein levels not only of DNA-PKcs, but also of tankyrase 1 and ATM. Consistent with our previous work [35], optimal loss of DNA-PKcs protein after siRNA knockdown occurred three days after transfection, at which time tankyrase 1 protein levels were not reduced (Figure S5); treatment with the DNA-PKcs inhibitor (Nu7026) also did not affect tankyrase 1 levels (data not shown). Furthermore, and consistent with other studies [36,37], we found that ATM protein levels were down-regulated in tandem with siRNA knockdown of DNA-PKcs protein at this late time (Figure S5), an effect shown to be mediated by reduction of DNA-PKcs mRNA [36].

The time courses of the knockdowns are particularly informative, as siRNA depletion of tankyrase 1 protein occurred much more rapidly (within 12 hr) than siRNA knockdown of DNA-PKcs protein (three days). Also, depletion of tankyrase 1 protein resulted in concurrent and rapid degradation of DNA-PKcs protein (observed at 12 hr) mediated by proteolytic - not mRNA - degradation. Consistent with this view, we found that ATM protein levels were not reduced by tankyrase 1 siRNA knockdown (Figure S6), in contrast to ATM depletion with DNA-PKcs mRNA-mediated knockdown (Figure S5).

### Tankyrase PARP activity is required for DNA-PKcs protein stability

**Figure 7.**
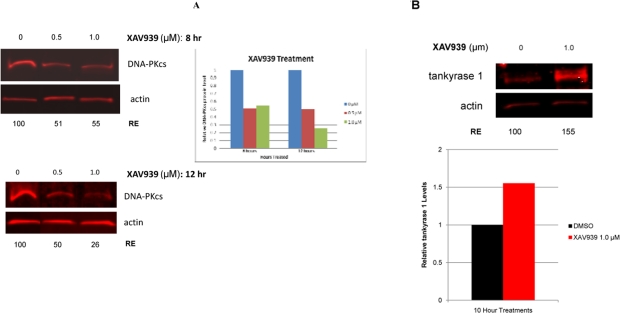
Inhibition of tankyrase PARP activity *decreases* DNA-PKcs and *increases* tankyrase 1 protein levels. (**A**) Treatment of human lymphoblasts with the tankyrase PARP domain inhibitor XAV939 (0.5 or 1.0 μM) for 8 hr reduced DNA-PKcs protein levels to ~50% of the relative DMSO control. XAV939 treatment (1.0 μM) for 12 hr reduced DNA-PKcs levels to approximately 25% of the relative DMSO control. (**B**) Tankyrase 1 levels increased over DMSO controls in cells treated with XAV939, providing evidence that DNA-PKcs protein stability relies on the catalytic function of tankyrase 1. (RE) Relative Expression.

To investigate possible protein-protein interaction, multiple protein complex immunoprecipitation (Co-IP) experiments were preformed, but they failed to demonstrate tight binding between tankyrase 1 and DNA-PKcs (data not shown), a negative result that argued against a physical interaction and for an enzymatic one. Initial support for tankyrase 1 enzymatic stabilization of DNA-PKcs protein was provided by treatments with the general PARP inhibitor 3-AB, which indicated that PARP activity is required for reducing IR-induced mutation frequencies (Figure 2). Additionally, 3-AB treatment alone (no tankyrase 1 knockdown) was sufficient to reduce DNA-PKcs protein levels (Figure S7), suggesting that the catalytic activity of tankyrase 1 is critical for DNA-PKcs stability. In support of the importance of tankyrase 1 PARsylating action for DNA-PKcs stability, treatment with the recently available small molecule inhibitor XAV939, which specifically inhibits tankyrase PARP activity at the concentrations selected [26], dramatically decreased DNA-PKcs protein levels (Figure 7), confirming the critical role of tankyrase poly-ADP-ribosylation activity in maintaining DNA-PKcs protein stability. Examination of various treatment times (2, 5, 8, 12, 18, 24 and 48 hr) and XAV939 concentrations (0.1, 0.5 or 1.0μM) revealed significant reduction of DNA-PKcs protein levels by 8 hours with exposures of either 0.5 μM or 1.0 μM XAV939 (Figure 7). The greatest reduction of DNA-PKcs protein levels (< 25% relative expression compared to DMSO treated controls) occurred at 12 hr with 1.0 μM XAV939 exposure. Later time points (24 hr) show that DNA-PKcs protein levels recover relative to DMSO treated controls, which may have been due to loss of inhibitor potency in culture (data not shown).

### Tankyrase 1 protein levels increase in response to tankyrase PARP inhibition

Inhibition of the tankyrase PARP domain with XAV939 did not diminish tankyrase 1 levels. To the contrary, treatment of human lymphoblasts with 1.0 μM XAV939 resulted in a significant increase of tankyrase 1 levels over DMSO treated controls (Figure 7). AutoPARsylation of tankyrase dissociates it from multimerized tankyrases in the growing pADPr chain [38], resulting in proteasome mediated degradation of tankyrase if not de-PARsylated [27]. Therefore, inhibiting tankyrase PARP activity blocks the ability of tankyrase 1 to auto-PARsylate, thereby shielding the protein from potential ubiquitination. These findings not only further support DNA-PKcs protein stability dependence on tankyrase-specific PARP activity, they also suggest that this catalytic activity, rather than tankyrase 1 protein levels per se, is what provides protection of DNA-PKcs since DNA-PKcs levels decrease despite elevated tankyrase 1. We speculate that tankyrase 1 PARsylates DNA-PKcs directly, resulting in a dynamic, yet consistent pool of PARsylated DNA-PKcs.

### DNA-PKcs protein levels decrease in response to PARG inhibition

**Figure 8.**
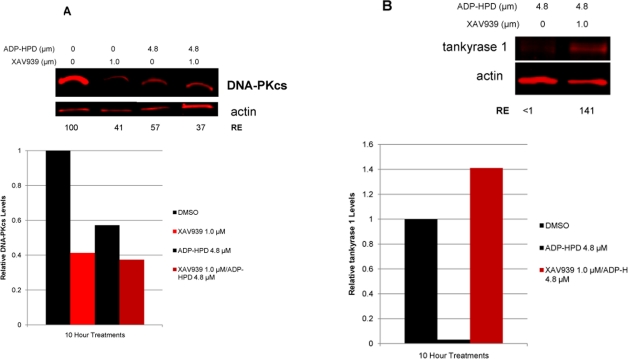
Inhibition of PARG activity reduces *both* DNA-PKcs and tankyrase 1 protein levels. (**A**) Treatment with the PARG inhibitor ADP-HPD (10 hr) prevents removal of pADPr from PARP modified proteins and resulted in reduction of DNA-PKcs levels relative to the DMSO treated control. The combination treatment of both ADP-HPD and XAV939 (10 hr) resulted in reduction of DNA-PKcs, similar to XAV939 treatment alone (Figure 7A). (**B**) PARG inhibition also decreased protein levels of tankyrase 1 compared to control. ADP-HPD/XAV939 combined treatment resulted in elevated tankyrase 1 levels, similar to XAV939 treatment alone (Figure 7B).

Degradation of ADP-ribose polymers is rapidly catalyzed by poly(ADP-ribose) glycohydrolase (PARG) [39]. To further explore tankyrase-dependent PARsylation of DNA-PKcs, we utilized the potent PARG inhibitor ADP-HPD [40]. We anticipated that inhibition of PARG activity would result in increased DNA-PKcs protein levels, as DNA-PKcs would become irreversibly PARsylated in a stable, proteasome resistant conformation. However, DNA-PKcs protein levels were diminished both with PARG inhibition alone and in combined ADP-HPD and XAV939 treatment (Figure 8). Probing the ADP-HPD treated lysate for tankyrase 1 protein revealed that PARG inhibition resulted in depletion of tankyrase 1, evidence of irreversible tankyrase autoPARsylation-dependent degradation (Figure 8). Thus, treatment with the PARG inhibitor ADP-HPD mimics tankyrase 1 siRNA knockdown in that both reduce tankyrase 1 protein levels, and so both also result in depletion of DNA-PKcs. The ADP-HPD/XAV939 combined treatment resulted in increased levels of tankyrase 1, suggesting that the inability to autoPARsylate predominates; i.e., there is little available to dePARsylate.

### Electrophoretic separation of PARsylated DNA-PKcs from unmodified pools of DNA-PKcs via SDS-PAGE

DNA-PKcs has been shown to be covalently modified by addition of poly(ADP-ribose) via PARP1 *in vitro*, resulting in a significant increase in DNA-PKcs kinase activity and suggesting a functional purpose for DNA-PKcs PARsylation [28]. Considering that our inhibitor studies cumulatively suggested covalent modification of DNA-PKcs via tankyrase 1-dependent PARsylation, we sought evidence of a high molecular weight pool of DNA-PKcs dependent upon tankyrase 1 catalytic PARP activity.

**Figure 9.**
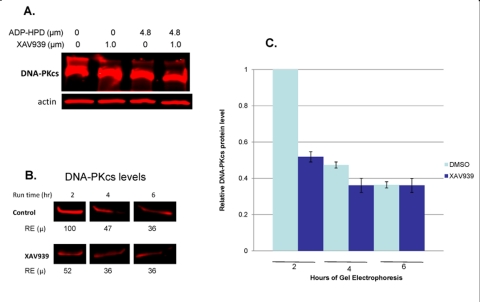
Identification of tankyrase-dependent high molecular weight forms of DNA-PKcs. (**A**) Over-exposed western blot reveals high molecular weight forms of DNA-PKcs, which are dependent upon the PARsylating function of tankyrase 1. A high molecular weight “smear” appears above the primary DNA-PKcs band in the untreated control, which is absent with XAV939 treatment; a lower molecular weight “smear” appears below the primary DNA-PKcs band with XAV939 treatment (degradation products). (**B** and **C**) Western blot analysis of same samples (not overexposed) and quantification of DNA-PKcs protein levels following 2, 4 and 6 hour SDS-PAGE run times for DMSO treated controls and XAV939 treated samples to separate high molecular weight forms. The control contains a large amount of DNA-PKcs in a modified, high molecular weight form, as seen by the reduction in DNA-PKcs band intensities over the longer run times (2hr compared to 4 and 6 hr run times; p ≤ 0.001). No significant reduction of DNA-PKcs band intensity over longer run times was observed in XAV939 treated samples (p > 0.05). RE (μ) are an average of two western blot analyses, which were used to calculate SEMs. P-values were determined by a *t* test to compare two means.

Gel electrophoresis facilitated visualization (upon overexposure) of a high molecular weight pool of DNA-PKcs present in DMSO treated controls, much of which resided in the loaded well (Figure 9). Further, treatments with XAV939, ADP-HPD and XAV939/ADP-HPD combined, resulted in diminution of this high molecular weight pool of DNA-PKcs, as well as a corresponding accumulation of DNA-PKcs degradation products compared to the DMSO treated controls (Figure 9). Due to the fact that the high molecular weight pool of DNA-PKcs was dependent upon catalytically active tankyrase (the only variable), we believe it represents PARsylated forms of DNA-PKcs. To further support this supposition, DMSO controls and XAV939-treated samples (8 hr) were independently loaded every 2 hours in individual wells of a gradient gel over 6 hours (2, 4, and 6 hr total run times). Here, our aim was to separate post-translationally modified, high molecular weight forms of DNA-PKcs from unmodified pools with run time. Upon quantification (gel not over-exposed) of the untreated control, a significant reduction of the primary DNA-PKcs protein band was observed between the 2 and 4 hour run times; i.e., at 4 hr, DNA-PKcs levels were reduced to ~50% relative to that detected at 2 hr (p = 0.001). Levels of DNA- PKcs detected at the 4 and 6 hr run times did not differ significantly (p > 0.05) (Figure 9). In contrast, the XAV939-treated samples lost little DNA-PKcs over the range of run times, indicating that a pool of tankyrase-dependent modified DNA-PKcs exists under normal conditions that is not present with tankyrase PARP inhibition. This result, together with the presence of a high molecular weight pool of DNA-PKcs in untreated controls, which was absent in XAV939 treated samples, supports the existence of a heterogeneous population of DNA-PKcs spanning a wide range of molecular weights, representing the various degrees of tankyrase 1-dependent PARsylated DNA-PKcs.

### Are the observed effects due to tankyrase 1, or DNA-PKcs?

We examined mutagenesis following DNA-PKcs siRNA knockdown and/or chemical inhibition of DNA-PKcs kinase activity (Nu 7026), and compared these with the effects of tankyrase 1 knockdown on MFs (Figure S8). For spontaneous mutagenesis, all of the conditions resulted in elevated background MFs compared to the mock-transfected control (all p < 0.001). We note that this is not always the case; e.g., background MFs were not significantly elevated in treated vs. mock controls in other experiments (Figure 2), possibly due to the fact that spontaneous mutants accumulate over a period of 4-10 days, depending on the details of a particular experiment, while a tankyrase 1 siRNA knockdown is effective for only a day or two. Therefore, the proportion of the total time in which spontaneous mutants accumulate under knockdown conditions could vary from 10% to 50%, making detection of a significant increase in background MF less problematic.

For IR-induced mutagenesis, all treatments to reduce levels of tankyrase 1 protein or to inhibit DNA-PKcs activity resulted in significant, and similar, increases in IR-induced mutation (all p < 0.05). (Figure S8) The combination of DNA-PKcs siRNA knockdown and inhibition with Nu 7026 was more effective at increasing radiation mutagenesis than was either treatment alone (p < 0.001, for 2 Gy of γ-rays or ^56^Fe). Also of interest was that the tankyrase 1 siRNA knockdown in combination with the DNA-PKcs inhibitor was more effective at increasing radiation mutagenesis than was either treatment alone (p ≤ 0.001). We believe that the significant elevation of the combined treatments at 2 Gy reflects the fact that not only are DNA-PKcs protein levels drastically reduced by virtue of either DNA-PKcs or tankyrase 1 siRNA knockdown, but in addition, what protein does remain has been rendered inactive by inhibition of its kinase activity.

The obligate reduction of DNA-PKcs protein that occurs with tankyrase 1 siRNA knockdown would also be expected to result in uncapping of telomeres produced by leading-strand synthesis [2]. While a significant increase in spontaneous telomere-telomere fusion events with tankyrase 1 depletion was not observed, telomere-DSB fusion events were evident [4,5] (Figure 3). Telomere-DSB fusion would be the most likely expected phenotype resulting from rapid, transient and only partial reduction of DNA-PKcs, as these events require only one uncapped telomere (not two as for telomere-telomere fusion). The increases in telomere-DSB fusion frequencies seen following IR exposure were similar with either tankyrase 1 depletion, DNA-PKcs inhibition or the two treatments combined (all p < 0.023). In regards to telomeric recombination, siRNA depletion of DNA-PKcs in normal human fibroblasts did not significantly elevate T-SCE levels (data not shown). Together, these results suggest that tankyrase 1 (not DNA-PKcs) is responsible for regulation of T-SCE frequencies, while DNA-PKcs (not tankyrase 1) participates in telomeric end-capping.

## DISCUSSION

### Tankyrase 1 regulates telomeric recombination (T-SCE)

Utilizing siRNA knockdown of tankyrase 1 in a variety of human cell lines, we found that its deficiency results in increased levels of telomeric recombination, visualized as T-SCE, in telomerase negative backgrounds. An especially appealing model invokes reduced levels of tankyrase 1 resulting in increased levels of the telomere repeat binding factor TRF1 remaining associated with telomeres [22]. This scenario would be expected to increase replication stress and fork stalling within the inherently challenging telomeric repeats [41,42], ultimately increasing T-SCE frequencies in order to “bypass” problems and continue replication. We originally suggested that in telomerase deficient backgrounds this ubiquitous SCE recombination-based mechanism might be used to advantage at telomeres to extend cellular proliferative life [11], however more recent work and modeling suggest that T-SCE possess the potential to accelerate cellular senescence, and so may be a contributing factor in premature aging [34,43]. It is also tempting to conjecture that failure and/or delay of resolution of sister telomere cohesion after replication in the setting of reduced tankyrase 1 [23] may serve to facilitate time and opportunity for T-SCE.

It has recently been reported that telomeres in telomerase positive cells deficient in tankyrase 1 become unprotected and undergo sister chromatid telomere fusion [44]. Of particular interest and relevance in this regard, are recent and elegant demonstrations that replication stress can induce sister-chromatid bridging at fragile sites in mitosis [45], and that mammalian telomeres resemble fragile sites [46], likely circumstances that may well contribute to sister telomeres not being properly or completely capped in the absence of tankyrase 1 and their subsequent vulnerability to cohesion and/or fusion.

Telomere dysfunction, defined here as failure to effectively cap telomeres, and not as critical shortening, is reminiscent of deprotected leading-strand telomeres in DNA-PKcs (and TRF2) deficient backgrounds at or shortly after replication, except that these telomere fusions occur between uncapped leading-strand telomeres of different chromatids [2,8], not between leading-lagging-strand telomeres of sister chromatids [44]. The obligate reduction of DNA-PKcs protein levels that we demonstrate here with siRNA depletion of tankyrase 1 would also be expected to result in uncapping of leading-strand telomeres. The relatively low frequency of chromatid-type telomere fusion we observed in DNA-PKcs deficient backgrounds, together with the incomplete, transient nature of the tankyrase 1 knockdown and associated DNA-PKcs degradation, likely explain the paucity of telomere-telomere fusion seen here. Even so, an increase in telomere-DSB fusion was observed with tankyrase 1 depletion following insult (exposure to IR), an observation consistent with our previous studies identifying and characterizing telomere uncapping in the context of DNA-PKcs deficiency [4,5]. The occurrence of telomere-DSB fusion is also consistent with our observation of increased chromosomal terminal deletions upon tankyrase 1 depletion (Figure 3), as some of these unrejoined terminal fragments, which by definition possess a telomere, become involved with IR-induced DSBs or vice-versa. Telomere uncapping, as well as unrejoining and/or misrejoining of DNA ends, are both occurrences promoted by DNA-PKcs deficiency; the end-joining that does occur in such backgrounds requires enlistment of backup pathways of NHEJ, which can be PARP-mediated [47]. Interestingly, it was recently suggested that DNA-PK normally acts to prevent back-up NHEJ from operating at telomeric ends [48].

### Tankyrase 1 regulates DNA repair via PARsylation-mediated stabilization of DNA-PKcs

**Figure 10.**
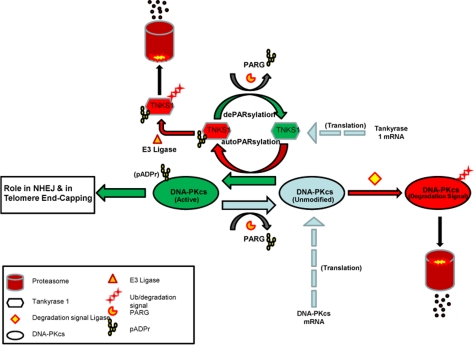
Model of DNA-PKcs existence in three dynamic pools; unmodified (blue), PARsylated (green), and marked for proteasome-mediated degradation (red). Unmodified pools of DNA-PKcs are PARsylated in a tankyrase 1 catalytic activity dependent manner (green arrow), a modification that can be reversed via PARG activity, resulting in unmodified DNA-PKcs (blue arrow). Once tankyrase 1 has PARsylated DNA-PKcs, tankyrase 1 auto-PARsylates (red) to dissociate from the multimerized-tankyrase complex. Tankyrase 1 relies on PARG activity to remove the auto-pADPr group and remain in an active state (green), otherwise, tankyrase 1 is ubiquitinated and targeted for degradation. The unmodified pool of DNA-PKcs is subject to being marked with a proteasome tag and subsequent degradation if not PARsylated by tankyrase 1. In conjunction with translation of new DNA-PKcs mRNA, there is a dynamic for DNA-PKcs PARsylation that shields a subpopulation of DNA-PKcs from degradation and perhaps represents the pool of kinase active DNA-PKcs. If this dynamic is disrupted by tankyrase 1 depletion or inhibition of its PARP catalytic activity, DNA-PKcs accumulates in the unmodified form and is forced to the right (degradation) resulting in depletion of DNA-PKcs protein.

Depletion of tankyrase 1 via siRNA transfection in various human cell lines resulted in increased sensitivity to IR-induced cell killing, mutagenesis and chromosome aberration, notably terminal deletion, end-points consistent with compromised DNA damage response/repair. However, knockdown or inhibition of tankyrase 1 also resulted in rapid reduction of DNA-PKcs protein levels, providing a likely explanation for the radiosensitivity and instability phenotypes observed. Tankyrase 1 siRNA depletion mediated DNA-PKcs decrease occurred much more quickly than it did after deliberate siRNA knockdown of DNA-PKcs, suggesting that DNA-PKcs protein reduction upon loss of tankyrase 1 does not proceed via an RNA pathway, but rather is due to loss of protein stability and subsequent proteolytic degradation, a scenario supported by our qRT-PCR and proteasome inhibitor studies, as well as by evaluation of ATM levels. Furthermore, we provide new mechanistic insight in that PARsylation activity is essential for DNA-PKcs stability, as both general and tankyrase specific PARP activity inhibition rapidly reduced DNA-PKcs protein levels. The small molecule inhibitor XAV939 specifically inhibits tankyrase-dependent PARP activity by binding the conserved catalytic domains of both tankyrase1 and tankyrase 2 [26]. The depletion of DNA-PKcs observed with XAV939 exposure strongly suggests that DNA-PKcs protein stability is reliant on PARsylation via tankyrase PARP activity specifically. Although XAV939 inhibits tankyrase 2 activity at lower concentrations than tankyrase 1 (IC50 = 0.004 and 0.011 μM respectively) [26], our tankyrase 1 siRNA studies provide key evidence supporting tankyrase 1 being responsible for DNA-PKcs stability, as tankyrase 2 mRNA levels were not impacted by tankyrase 1 siRNA treatment; i.e., tankyrase 2 mRNA levels remained high, yet DNA-PKcs protein still plummeted (Figure 5). Taken together with the reciprocal actions of PARG, our results demonstrate that DNA-PKcs protein stability is dependent on the PARsylating activity of tankyrase 1 for protection from proteasome-mediated degradation. We provide supporting evidence that, in conjunction with the findings of others [28,38], leads us to propose a model in which DNA-PKcs exists in three pools; unmodified, PARsylated (active), and marked for proteasome-mediated degradation (Figure 10).

In addition to the demonstration that PARsylation of DNA-PKcs stimulates its kinase activity [28], and the identification of DNA-PKcs as an poly(ADP-ribose)-associated protein [29], other interactions between PARP1 and DNA-PKcs have been reported, including reciprocal regulation of their enzymatic activities (PARsylation of DNA-PKcs and phosphorylation of PARP1), and inhibition of DNA-PKcs kinase activity by inactive PARP [49]. Here, we demonstrate that depletion of the telomeric PARP tankyrase 1 results in rapid loss of DNA-PKcs protein stability, providing the first evidence for tankyrase 1 - DNA-PKcs interaction and its functional significance. Interestingly, both gain and loss of tankyrase 1 protein resulted in DNA-PKcs deficiency, and so either would be expected to contribute to carcinogenesis. In fact, tankyrase 1 protein levels have been shown to be downregulated in colon cancer [50], while overexpression of tankyrase 1 has been observed in breast cancer [51], both of which likely reflect loss of tankyrase 1 PARP activity.

Our investigation reveals not only that the damage response observed upon depletion of the telomeric PARP tankyrase 1 likely results from reduced levels of the NHEJ protein DNA-PKcs (via loss of PARsylation mediated protection from proteolytic degradation), it also provides additional support of tankyrase 1 as a therapeutic target [52]. Altered levels of tankyrase 1, whether resulting from individual polymorphisms and/or intentional inhibition of tankyrase 1, would be expected to compromise DNA damage repair by DNA-PK mediated NHEJ, so such a therapeutic strategy may be especially effective when combined with radiation therapy or in some tumor types, for example BRCA1/2 associated breast cancers [53], perhaps in similar fashion to the encouraging therapeutic strategy of using PARP inhibitors against cancers associated with BRCA1/2 mutation [54]. The demonstration that inhibition of PARP activity provided specific anti-tumor activity toward BRCA2 deficient tumors was the first time DNA repair had been exploited to kill a cancer [55]. Interestingly, PARP [56,57], and DNA-PKcs [58] have been shown to interact/cooperate with WRN, the protein mutated in the premature aging Werner syndrome, raising the possibility of tankyrase 1 specific involvement in aging. Such a concept is supported by the elevated T-SCE frequencies we report here, in addition to tankyrase 1 involvement in the other age-related processes of telomere maintenance, and as we also demonstrate, DNA repair, another capability shown to decline with age. These studies reveal a new facet of the intriguing inter-relationships between telomeres and DNA repair, which have important implications in both cancer and aging, as well as highlight that there is much to learn from how cells deal with DNA ends.

## MATERIALS AND METHODS

### Cell lines

Characterization of telomerase activity during spontaneous immortalization of Li-Fraumeni syndrome skin fibroblasts [MDAH087 (087) telomerase negative (ALT) and MDAH041 [41] telomerase positive] has been described previously [33]. The mutant p53 status of these cell lines favored evaluation of telomere dysfunction and MDAH087 provided an ALT background. Telomerase negative (not ALT), normal neonatal 5C human dermal fibroblasts (HDFn; Cascade Biologics) were used at low passage, maintain-ed in α-MEM medium (Hyclone) supplemented with 10% fetal calf serum (Sigma Aldrich) and 1% pen-strep (Hyclone), and incubated at 37oC in an atmosphere of 95% air and 5% carbon dioxide. A telomerase positive background was evaluated in the hTERT-immortalized human foreskin fibroblast cell line BJ-5ta (ATCC), which was sustained similarly.

WTK1 human lymphoblastoid cells have a stable karyotype (47, X, Y 13+, 14q+) and were derived from the WI-L2 line [59]. WTK1 cells were used for mutation analyses as they are heterozygous at the thymidine kinase locus; they also have a single amino acid substitution in codon 237 at TP53. WTK1 cells were maintained in RPMI1640 medium (Hyclone) supplemented with 10% heat-inactivated horse serum (Sigma-Aldrich) and 1% pen-strep (Hyclone).

### Tankyrase 1 siRNA knockdown

The following siRNA sequences were used for the targeted silencing of tankyrase 1 (Dharmacon Research) and DNA-PKcs (Qiagen): tankyrase 1 siRNA1: 5' AGG AAG GAG ACA CAG AUA UdTdT 3'; tankyrase 1 siRNA2: 5' CCU GGA AGU AGC UGA AUA UdTdT 3'; DNA-PKcs siRNA: 5'-GAUCGCACCUUACUCUGUUdTdT -3'. WTK1 lymphoblasts were seeded in RPMI 1640 medium with 5% horse serum (no antibiotics), at a concentration of 5x105 cell/ml, 20 hr prior to transfection. The 5C human dermal fibroblasts were seeded at 50-60% confluency in α-MEM medium with 10% fetal bovine serum (no antibiotics), one day prior to transfection. Cells were transfected with tankyrase 1 or DNA-PKcs siRNA (20nM) using Lipofectamine 2000 and OptiMEM (Invitrogen) serum free media; in some cases, a second transfection was done 24 hr later to maintain knockdown. The mock sample included in every experiment, contained only Lipofectamine 2000 with OptiMEM and no siRNA. Cells were harvested at various times post siRNA transfection and processed for Western blot analysis, or used in experiments to assess radiation-induced effects.

### Western blot analysis

Western blot analysis was always performed to confirm successful knockdown of target protein level before proceeding with evaluation of endpoints (representative blots shown in S1). Cells were harvested, centrifuged and resuspended in cold PBS (without Mg+ Ca+) twice, then immersed in 1x RIPA buffer (1x TBS, 1% Nonidet P-40, 0.5% sodium deoxycholate, 0.1% SDS, 0.004% sodium azide) and protease inhibitor cocktail (Santa Cruz Biotechnology), incubated on ice for 5-10 min, then passed through a 25 gauge syringe needle and centrifuged for 10 min at 140,000x g at 4°C. Protein in the supernatant was quantified using a BSA protein assay. Thirty-five to 50μg of the supernatant proteins were fractionated by SDS-PAGE (Bio-Rad) and transferred to Immobilon-FL PVDF membranes (Millipore). Blots were blocked in 5% skim milk or 5% BSA in TBS containing 0.1% Tween 20 and incubated overnight at 4°C with the following primary antibodies: rabbit polyclonal anti-tankyrase 1 (200 μg/ml; Santa Cruz Biotechnology); mouse monoclonal anti-actin (200 μg/ml; Santa Cruz Biotechnology); mouse monoclonal anti-PKcs Ab-4 (200 μg/ml; Neomarker); rabbit polyclonal anti-ATM (1mg/ml; Abcam). The blots were washed three times with TBS containing 0.1% Tween 20 and incubated with secondary antibody 680IRDye-conjugated goat polyclonal anti-rabbit IgG or IRDye 800CW-conjugated goat polyclonal anti-mouse IgG (1:15,000; LI-COR Biosciences). Bound antibodies were detected and using an Odyssey fluorescent imaging system (LI-COR Biosciences); blots were quantified according to manufacturers' instructions and normalized to independent actin loading controls.

Quantification of some blots was accomplished by importing images into Photoshop CS3 and analyzing as per a protocol adapted from the National Institutes of Health (http:/rsb.info.nih.gov/ij/index.html). Analysis involved first, multiplying the mean measured value by the number of pixels to obtain an “absolute intensity” value, an integrated measure of intensity and size of bands. Next, the relative intensity for each sample band was calculated by dividing the absolute intensity of each band by the absolute intensity of the standard (the mock transfection sample).

### Chemical inhibition

Nu7026 (Sigma-Aldrich), a competitive and highly selective inhibitor of DNA-PKcs kinase activity, was added to WTK1 cultures after siRNA transfection at a final concentration of 9 μM [49,60], and remained on samples until collected for mutagenesis or cytogenetic analyses. We have consistently found that this concentration of Nu7026 yields similar results for these end points as does siRNA knockdown of DNA-PKcs.

3-aminobenzamide (3-AB; Sigma-Aldrich) was used to inhibit global PARP activity at final concentrations ranging from 10 and 100 μM, to 10 and 20 mM. 3-AB was added to WTK1 cultures 24 hr prior to irradiation (or sham), which were then collected for mutation or western blot analyses and quantified.

XAV939, the recently identified small molecule shown to specifically inhibit PARP activity of tankyrase 1 (and tankyrase 2 at higher concentrations) [26], was used here at much lower concentrations than 3-AB. The tankyrase specific inhibitor XAV939 (Tocris) was solubilized in DMSO at 55°C to a stock concentration of 10mM, which was diluted to a working concentration of 100μM; final concentrations of 0.5μM or 1μM were well within the concentration parameters suggested for cell culture experiments to inhibit tankyrase specifically. Cultures were maintained under these conditions for the duration of the designated time course. Controls were exposed to DMSO alone. Following treatment, cells were lysed and prepared for western blot analysis. Tankyrase 1 and DNA-PKcs protein levels were normalized to the β-actin loading controls and quantified via LI-COR Odyssey software.

### MG132

WTK1 lymphoblasts were transfected with tankyrase 1 siRNA, or treated with 1.0 μM XAV939, then incubated with the proteosome inhibitor MG-132 (12.5 μM; Sigma-Aldrich) for 2 hr time windows starting at 8, 10, 12 or 24 hours after transfection [61]. Cell samples were harvested 4 hours after treatment for western blot analysis.

### ADP-HPD

WTK1 lymphoblasts were treated with the PARG inhibitor ADP-HPD [40] at 1.2 μM (EMD Chemicals) every 2.5 hours for a period of 10 hours, either alone or concurrently with XAV939 (1.0 μM final), at a final concentration of 4.8 μM ADP-HPD. Samples were harvested at 10 hours following the respective treatment and lysates were prepared for western blot analysis and quantified using the actin loading control for normalization.

### Electrophoretic separation of high molecular weight DNA-PKcs

WTK1 lymphoblasts treated with either DMSO or 1.0 μM XAV939 for 8 hours were loaded into independent wells of a 4-20% gradient SDS-PAGE every 2 hours over the course of 6 hours. At each time point, DMSO and XAV939 samples were loaded into wells immediately adjacent to the prior time point. The corresponding load times at 0, 2 and 4 hours resulted in total run times of 2, 4 and 6 hours respectively. Following completion of the final run time, the gel was analyzed via western blot for DNA-PKcs and normalized to actin loading controls, then quantified.

### Co-immunoprecipitation (Co-IP)

A Thermo Scientific Pierce Co-IP kit was used according to manufactures instructions to isolate native protein complexes from cell lysates by directly immobilizing purified antibody onto an agarose support. The following primary antibodies were used; rabbit polyclonal anti-tankyrase 1 (Santa Cruz Biotechnology) and mouse monoclonal anti-PKcs Ab-1 (Neomarker/Thermo Scientific).

### Irradiations

WTK1 lymphoblasts or 5C dermal fibroblasts were exposed to various doses of 137Cs γ-rays in a Mark I irradiator (J.L. Shepherd) located at Colorado State University, or to 1 GeV/n ^56^Fe (high Z high energy; HZE) particles at the NASA Space Radiation Laboratory at Brookhaven National Laboratory (NSRL/BNL).

### Mutation assay

WTK1 lymphoblasts were treated with CHAT (10-5 M 2'-deoxycytidine, 2 × 10-4 M hypoxanthine, 2 × 10-7 M aminopterin, 1.75 × 10-5 M thymidine; Sigma) for two days and CHT (CHAT without aminopterin) for one day to eliminate pre-existing TK- mutants. Following CHAT treatment, cells were transfected with tankyrase 1 siRNA and/or treated with Nu7026 or 3-AB. Three days later, cells were irradiated with γ-rays or HZE particles. Two days after irradiation, when phenotypic expression of newly induced mutants was complete, the mutant fractions (MF) were determined by plating in 96 well dishes. For plating efficiency, one cell/well was seeded, or for scoring mutants, 2000 cells/well were seeded in the presence of 2μg/ml trifluorothymidine (TFT; Sigma-Aldrich). Fresh TFT was added 11 days after plating, and plates were scored for positive or negative wells after 20 days. The MFs were calculated using the Poisson distribution [62] and statistical analyses were done by t-tests using Sigma Stat 3.5 (Systat Software).

### Surviving fraction assay

Two hours before exposure, exponentially growing cells were seeded into 60 mm dishes at various densities depending on the radiation dose to be delivered. After irradiation, plates were incubated at 37°C for 14-20 days in normal growth medium to allow for colony formation. Plates were rinsed, fixed with methanol, and stained with methylene blue. Colonies with >50 cells were counted and absolute plating efficiencies calculated for each dose. Surviving fractions represent the plating efficiency for the treated culture divided by the untreated control.

### Cytogenetic analyses

Chromosome-Orientation Fluorescence in situ hybridization (CO-FISH) was performed as previously described [7,35] with some modification. Following irradiation, cell cultures were incubated for various times, trypsinized and sub-cultured into medium containing the thymidine analog 5-bromo-2-deoxyuridine (BrdU, 10μM; Sigma-Aldrich) for one cell cycle. Slides were air dried and stained with Hoechst 33258 (0.50ng/μl; Sigma-Aldrich) for 15 minutes and exposed to 365 nm UV light (Stratalinker 2400) for 25 minutes. Following UV exposure, BrdU incorporated strands were digested with Exonuclease III (3U/μl in provided reaction buffer; Promega) at room temperature for 10 minutes. A Cy-3 conjuated (TTAGGG)3 PNA telomere probe (0.2μg/ml; Applied Biosystems) was hybridized at 37°C for 1.5 hr. Slides were rinsed in 70% formamide at 32°C for 10 min and dehydrated in another ethanol series before re-probing at 37°C for two hr. Following the second hybridization, slides were rinsed with 70% formamide at 32°C for 15 min followed by 5 min rinse in PN Buffer. Chromosomes were counterstained with DAPI (4,6-Diamidine-2-phenylindole dihydrochloride; Vectashield, Vector Laboratories). Preparations were examined and images captured and analyzed using a Zeiss Axioskop2 Plus microscope equipped with a Photometrics Coolsnap ES2 camera and Metavue 7.1 software.

### Scoring Criteria

T-SCE were scored as a CO-FISH telomere signal split between the two chromatids of a metaphase chromosome, which were often of unequal intensity due to unequal SCE [11]. G-SCE were scored on cells that had progressed through two rounds of replication in the presence of the BrdU; characteristic FPG harlequin staining was visualized using a mouse monoclonal anti-BrdU conjugated to Alexafluor 488 (FITC; Invitrogen) after CO-FISH treatment.

Telomere fusion necessitates that telomeres of adjoining chromosomes/chromatids fuse into a single CO-FISH signal and the DAPI signal remain continuous [2]. Telomere-DSB fusion appears as single-sided (on only one chromatid of a mitotic chromosome) interstitial blocks of CO-FISH telomere signal [4,5]. Chromo-some aberration frequencies (dicentrics, rings, terminal deletions, etc) were scored according to standard and accepted practice. Statistical analyses by Chi-square or Fisher's exact test (Sigma Stat 3.5; Systat Software) was done to determine significance.

### Quantitative Real-Time PCR (RT-PCR)

Alpha-MEM media (no antibiotics; Hyclone) was added to 5C human dermal fibroblasts (~50% confluent) 24 hrs prior to transfection of tankyrase 1 siRNA1 with Lipofectamine RNAiMAX Reagent (Invitrogen). Following transfection, α-MEM (no FBS, no antibiotic) was added to the flasks. Cells were harvested at 4, 8, 12, 18, 24 and 48 hours post transfection, and RNA was extracted using the RNeasy kit (Qiagen) with the optional on-column DNase treatment (Qiagen). RNA was subjected to electrophoresis to affirm integrity and assure no genomic DNA contamination. A mock transfection (lipofectamine, no siRNA) was done for each time point.

Quantitative RT-PCR analysis was used to evaluate mRNA transcript levels of tankyrase 1, tankyrase 2, and DNA-PKcs, relative to the housekeeping gene transferrin receptor C (TFRC). Total RNA extracted for each time point was used for reverse transcription reactions using the Verso cDNA kit (Abgene). The RT-PCR was performed using ABsolute SYBR Green Fluorescein mix (Abgene) with a total cDNA concentration of 54ng/reaction. The primers used to detect specific gene transcripts were as follows:

tankyrase 1 forward, 5'-TTGCTCTTTCCAACACAA GC-3' tankyrase 1 reverse, 5'-TACAGAACCACACGCTCC TC-3' tankyrase 2 forward, 5'-TCTTCAGGTCCATCTAGC CC-3' tankyrase 2 reverse, 5'-AAGCACCCTCTGTTCCAC TT-3' DNA-PKcs forward, 5'-AGCAAATGCACCGTTGTG GT-3' DNA-PKcs reverse, 5'-TCCTTCTTCAGGAGCTTCC A-3' TFRC forward, 5'-CGCTGGTCAGTTCGTGATTA-3' TFRC reverse, 5'-GCATTCCCGAAATCTGTTGT-3'.

Each sample was analyzed in triplicate for each transcript evaluated. Relative transcript analyses were done using the delta-delta Ct method where expression is determined relative to the controls at each time point [63]. Three independent RT-PCR runs were evaluated for statistical significance via the SAS System MEANS Procedure to generate means, standard deviations and standard error of the means for comparisons of each gene at each time point. Statistical analyses were performed using GraphPad Prism software. Figures containing three or more means were analyzed using ANOVA. When means differed significantly (p<0.05), Tukey's post hoc test was employed.

## SUPPLEMENTAL FIGURES


