# Supplementary material for: Regulatory roles of tankyrase 1 at telomeres and in DNA repair: suppression of T-SCE and stabilization of DNA-PKcs
Source: Aging (Albany NY). 2010 Oct 20;2(10):691–708. doi: 10.18632/aging.100210 (PMC2993799; doi:10.18632/aging.100210)
Supplement: Supplementary file 1 [file aging-02-691-s001.ppt]

## Slide 1
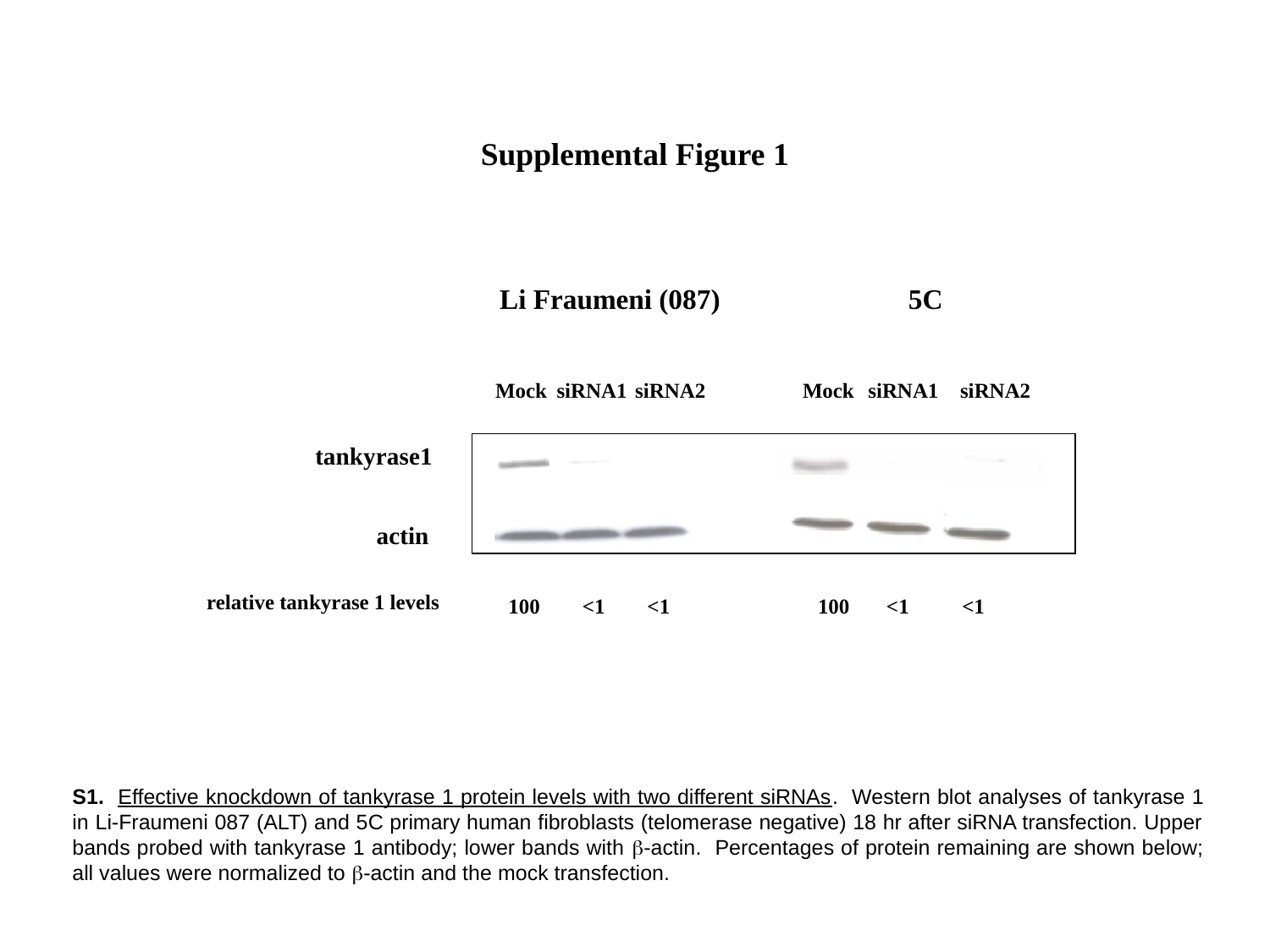

Supplemental Figure 1
Li Fraumeni (087)
5C
Mock
siRNA1
siRNA2
Mock
siRNA1
siRNA2
tankyrase1
actin
relative tankyrase 1 levels
100 <1 <1 100 <1 <1
S1. Effective knockdown of tankyrase 1 protein levels with two different siRNAs. Western blot analyses of tankyrase 1 in Li-Fraumeni 087 (ALT) and 5C primary human fibroblasts (telomerase negative) 18 hr after siRNA transfection. Upper bands probed with tankyrase 1 antibody; lower bands with β-actin. Percentages of protein remaining are shown below; all values were normalized to β-actin and the mock transfection.

## Slide 2
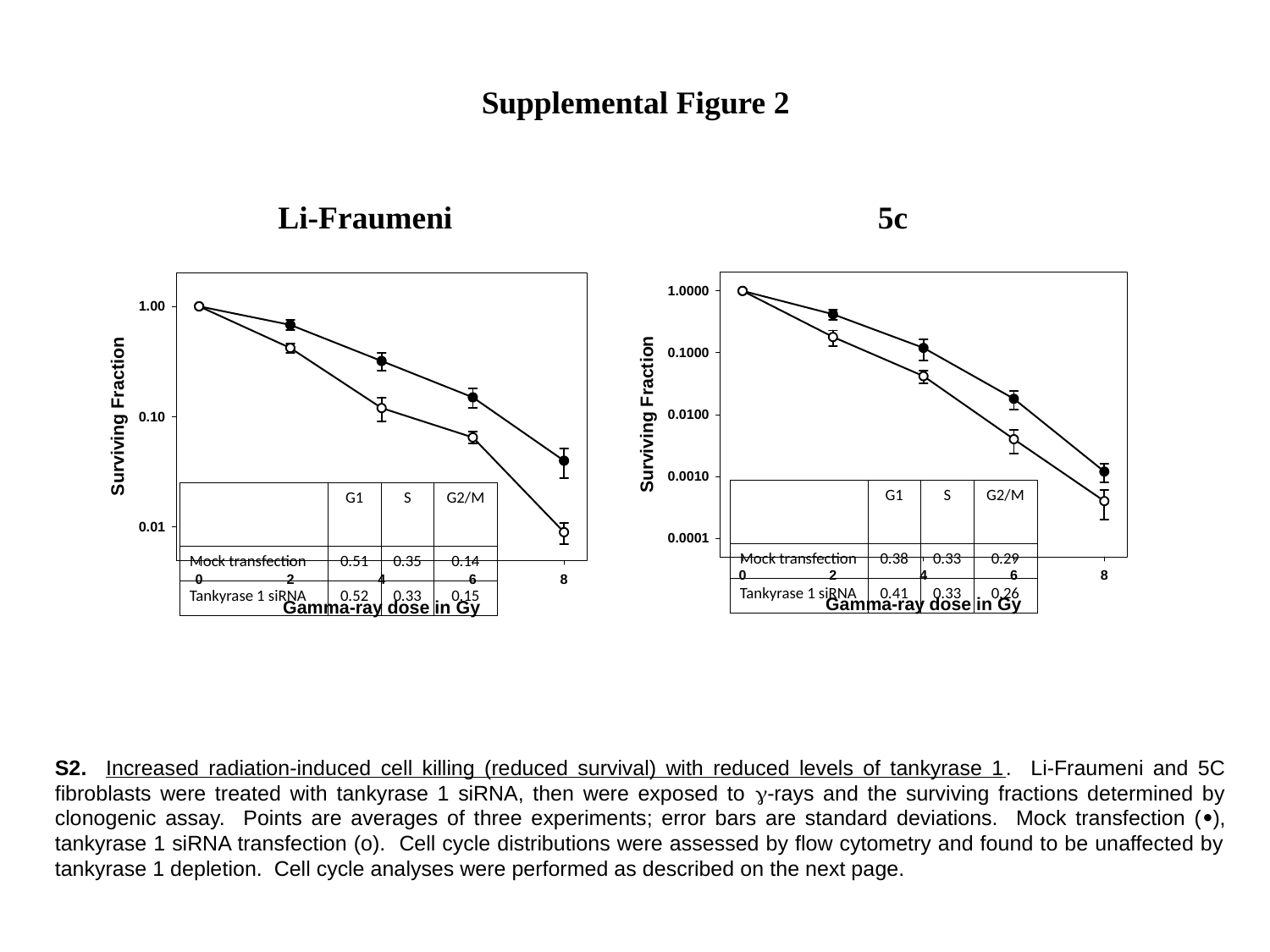

Supplemental Figure 2
Li-Fraumeni
5c
| | G1 | S | G2/M |
| --- | --- | --- | --- |
| Mock transfection | 0.38 | 0.33 | 0.29 |
| Tankyrase 1 siRNA | 0.41 | 0.33 | 0.26 |
| | G1 | S | G2/M |
| --- | --- | --- | --- |
| Mock transfection | 0.51 | 0.35 | 0.14 |
| Tankyrase 1 siRNA | 0.52 | 0.33 | 0.15 |
S2. Increased radiation-induced cell killing (reduced survival) with reduced levels of tankyrase 1. Li-Fraumeni and 5C fibroblasts were treated with tankyrase 1 siRNA, then were exposed to γ-rays and the surviving fractions determined by clonogenic assay. Points are averages of three experiments; error bars are standard deviations. Mock transfection (), tankyrase 1 siRNA transfection (o). Cell cycle distributions were assessed by flow cytometry and found to be unaffected by tankyrase 1 depletion. Cell cycle analyses were performed as described on the next page.

## Slide 3
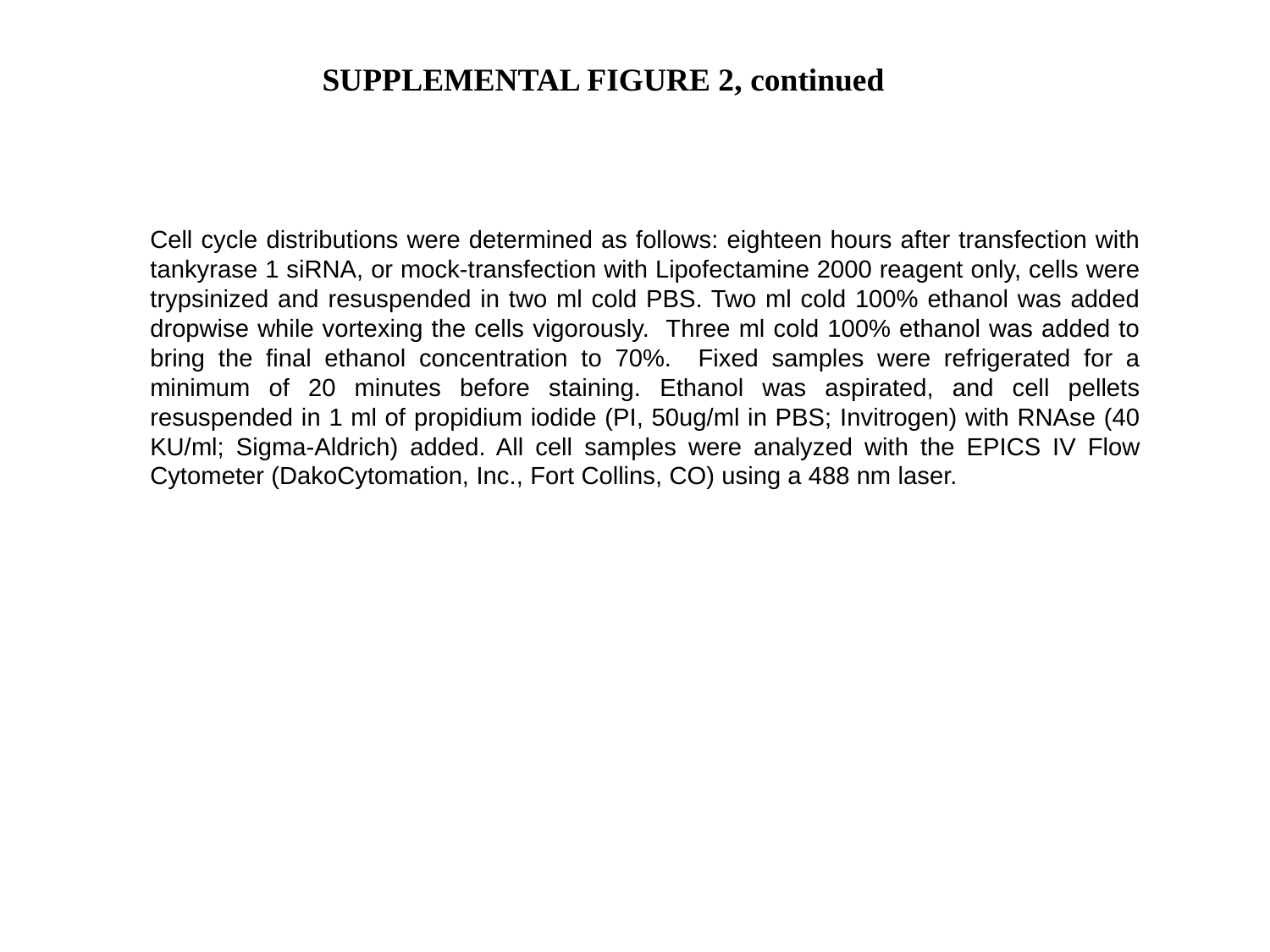

SUPPLEMENTAL FIGURE 2, continued
Cell cycle distributions were determined as follows: eighteen hours after transfection with tankyrase 1 siRNA, or mock-transfection with Lipofectamine 2000 reagent only, cells were trypsinized and resuspended in two ml cold PBS. Two ml cold 100% ethanol was added dropwise while vortexing the cells vigorously. Three ml cold 100% ethanol was added to bring the final ethanol concentration to 70%. Fixed samples were refrigerated for a minimum of 20 minutes before staining. Ethanol was aspirated, and cell pellets resuspended in 1 ml of propidium iodide (PI, 50ug/ml in PBS; Invitrogen) with RNAse (40 KU/ml; Sigma-Aldrich) added. All cell samples were analyzed with the EPICS IV Flow Cytometer (DakoCytomation, Inc., Fort Collins, CO) using a 488 nm laser.

## Slide 4
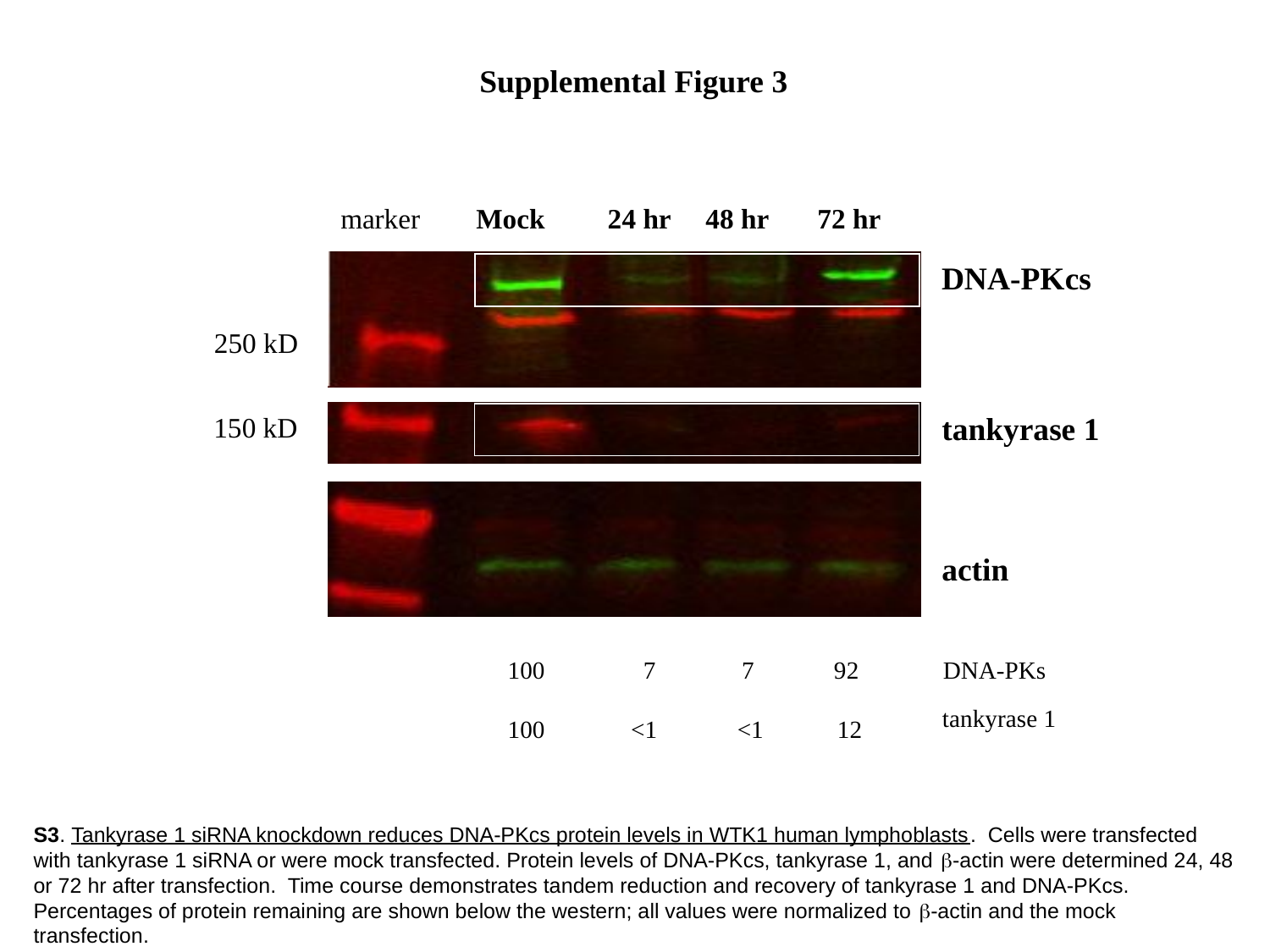

Supplemental Figure 3
 marker Mock 24 hr 48 hr 72 hr
DNA-PKcs
tankyrase 1
actin
250 kD
150 kD
100 7 7 92
 DNA-PKs
tankyrase 1
100 <1 <1 12
S3. Tankyrase 1 siRNA knockdown reduces DNA-PKcs protein levels in WTK1 human lymphoblasts. Cells were transfected with tankyrase 1 siRNA or were mock transfected. Protein levels of DNA-PKcs, tankyrase 1, and β-actin were determined 24, 48 or 72 hr after transfection. Time course demonstrates tandem reduction and recovery of tankyrase 1 and DNA-PKcs. Percentages of protein remaining are shown below the western; all values were normalized to β-actin and the mock transfection.

## Slide 5
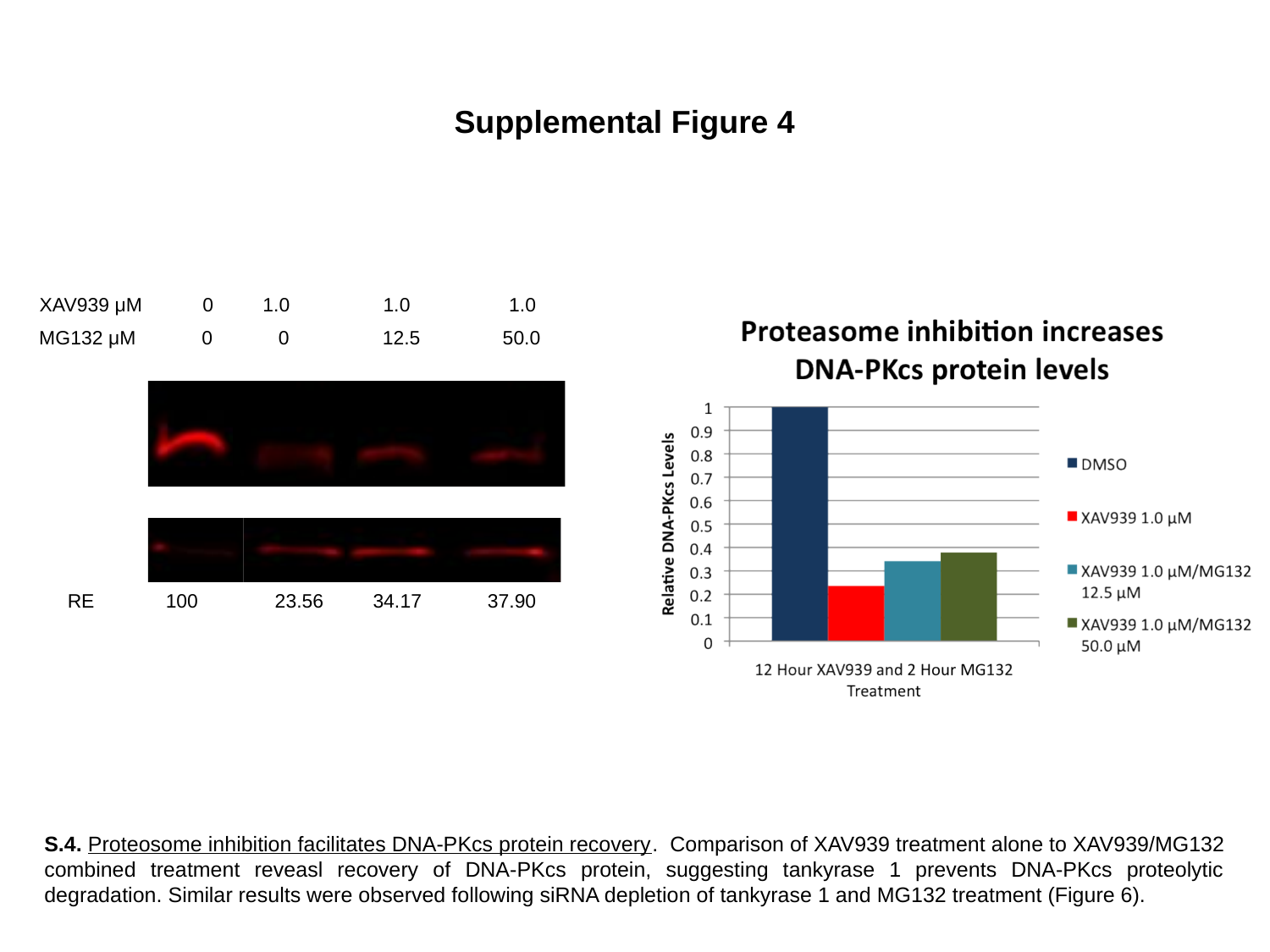

Supplemental Figure 4
 XAV939 μM 0 1.0 1.0 1.0
 MG132 μM 0 0 12.5 50.0
 RE 100 23.56 34.17 37.90
S.4. Proteosome inhibition facilitates DNA-PKcs protein recovery. Comparison of XAV939 treatment alone to XAV939/MG132 combined treatment reveasl recovery of DNA-PKcs protein, suggesting tankyrase 1 prevents DNA-PKcs proteolytic degradation. Similar results were observed following siRNA depletion of tankyrase 1 and MG132 treatment (Figure 6).

## Slide 6
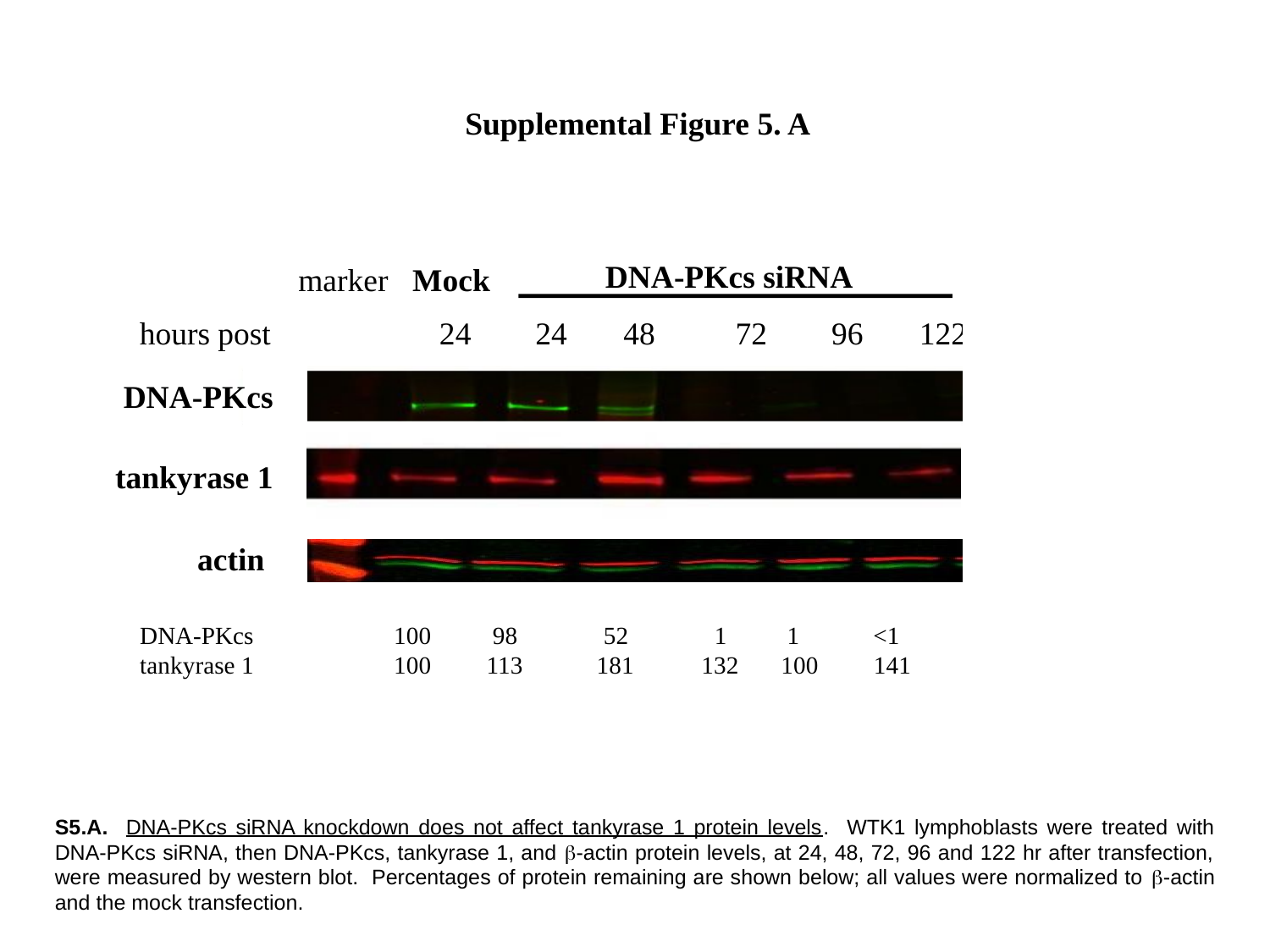

Supplemental Figure 5. A
DNA-PKcs siRNA
marker Mock
hours post 24 24 48 72 96 122
DNA-PKcs
tankyrase 1
actin
DNA-PKcs 	100 98 52 1 	 1 <1
tankyrase 1 	100 113 181 132 	 100 141
S5.A. DNA-PKcs siRNA knockdown does not affect tankyrase 1 protein levels. WTK1 lymphoblasts were treated with DNA-PKcs siRNA, then DNA-PKcs, tankyrase 1, and β-actin protein levels, at 24, 48, 72, 96 and 122 hr after transfection, were measured by western blot. Percentages of protein remaining are shown below; all values were normalized to β-actin and the mock transfection.

## Slide 7
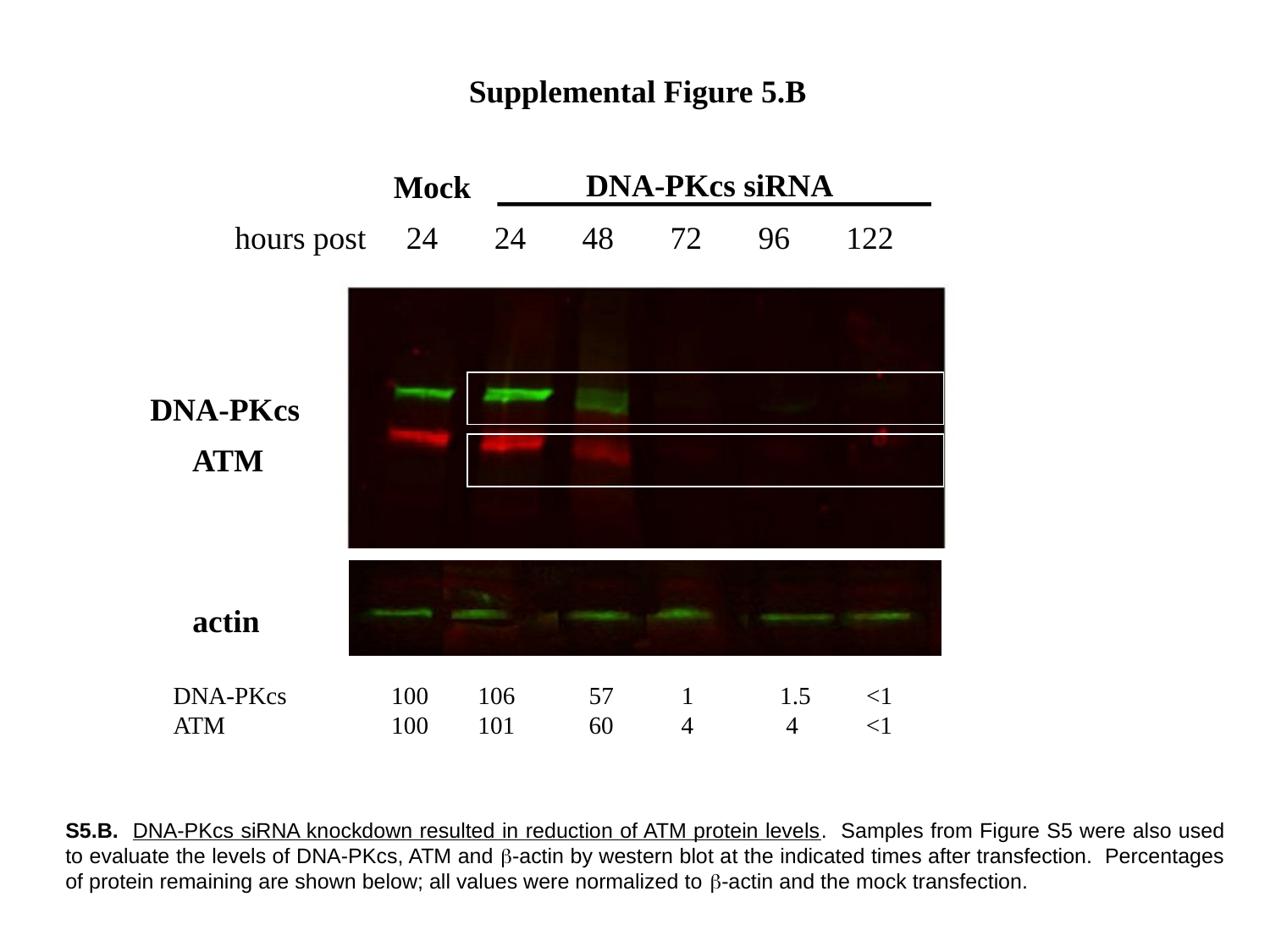

Supplemental Figure 5.B
DNA-PKcs siRNA
Mock
hours post 24 24 48 72 96 122
DNA-PKcs
ATM
actin
DNA-PKcs 100 106 57 1 1.5 <1
ATM 100 101 60 4 4 <1
S5.B. DNA-PKcs siRNA knockdown resulted in reduction of ATM protein levels. Samples from Figure S5 were also used to evaluate the levels of DNA-PKcs, ATM and β-actin by western blot at the indicated times after transfection. Percentages of protein remaining are shown below; all values were normalized to β-actin and the mock transfection.

## Slide 8
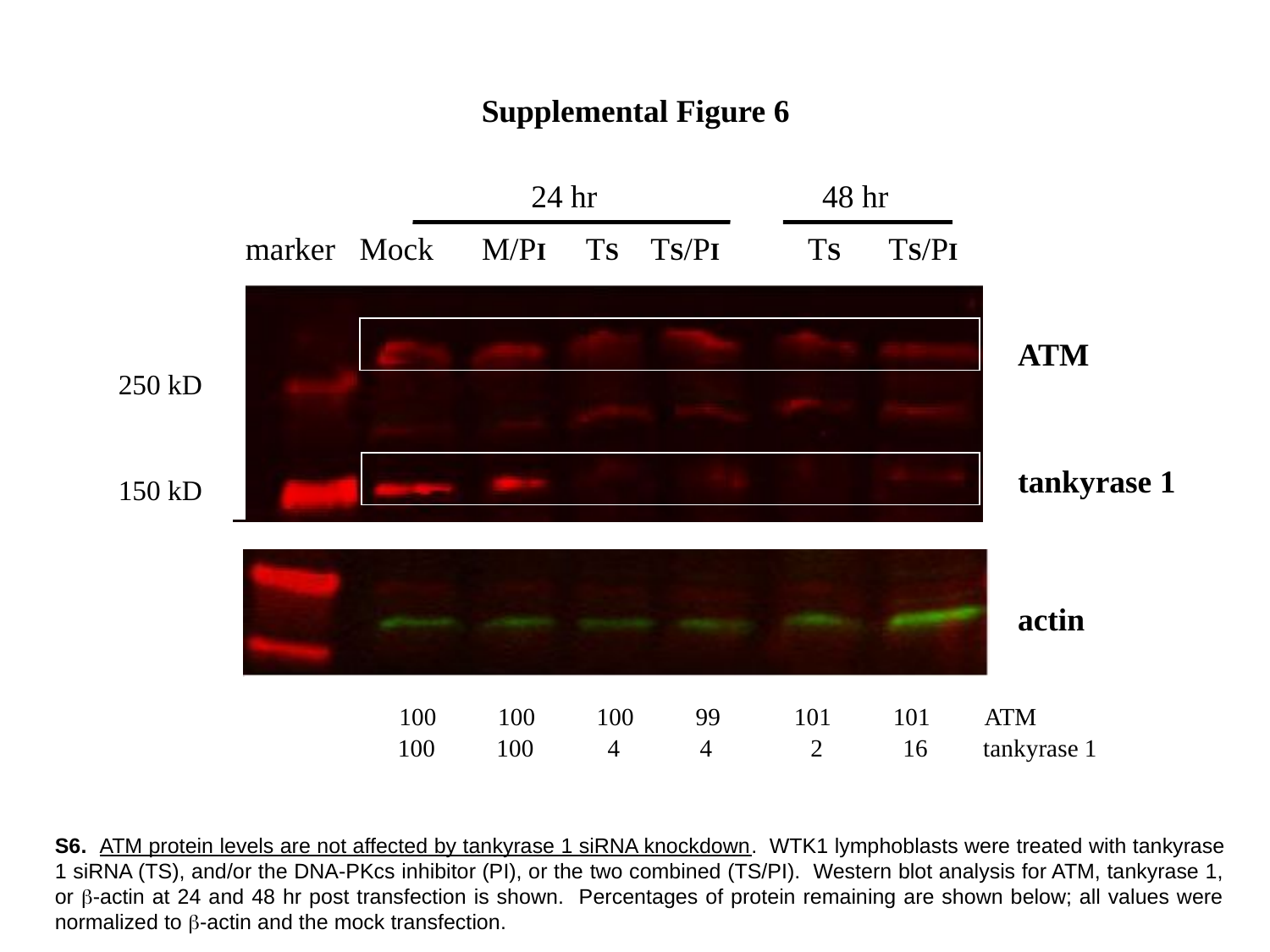

Supplemental Figure 6
24 hr 48 hr
marker Mock M/PI TS TS/PI TS TS/PI
ATM
250 kD
150 kD
actin
 100 100 100 99 101 101 ATM
 100 100 4 4 2 16 tankyrase 1
tankyrase 1
S6. ATM protein levels are not affected by tankyrase 1 siRNA knockdown. WTK1 lymphoblasts were treated with tankyrase 1 siRNA (TS), and/or the DNA-PKcs inhibitor (PI), or the two combined (TS/PI). Western blot analysis for ATM, tankyrase 1, or β-actin at 24 and 48 hr post transfection is shown. Percentages of protein remaining are shown below; all values were normalized to β-actin and the mock transfection.

## Slide 9
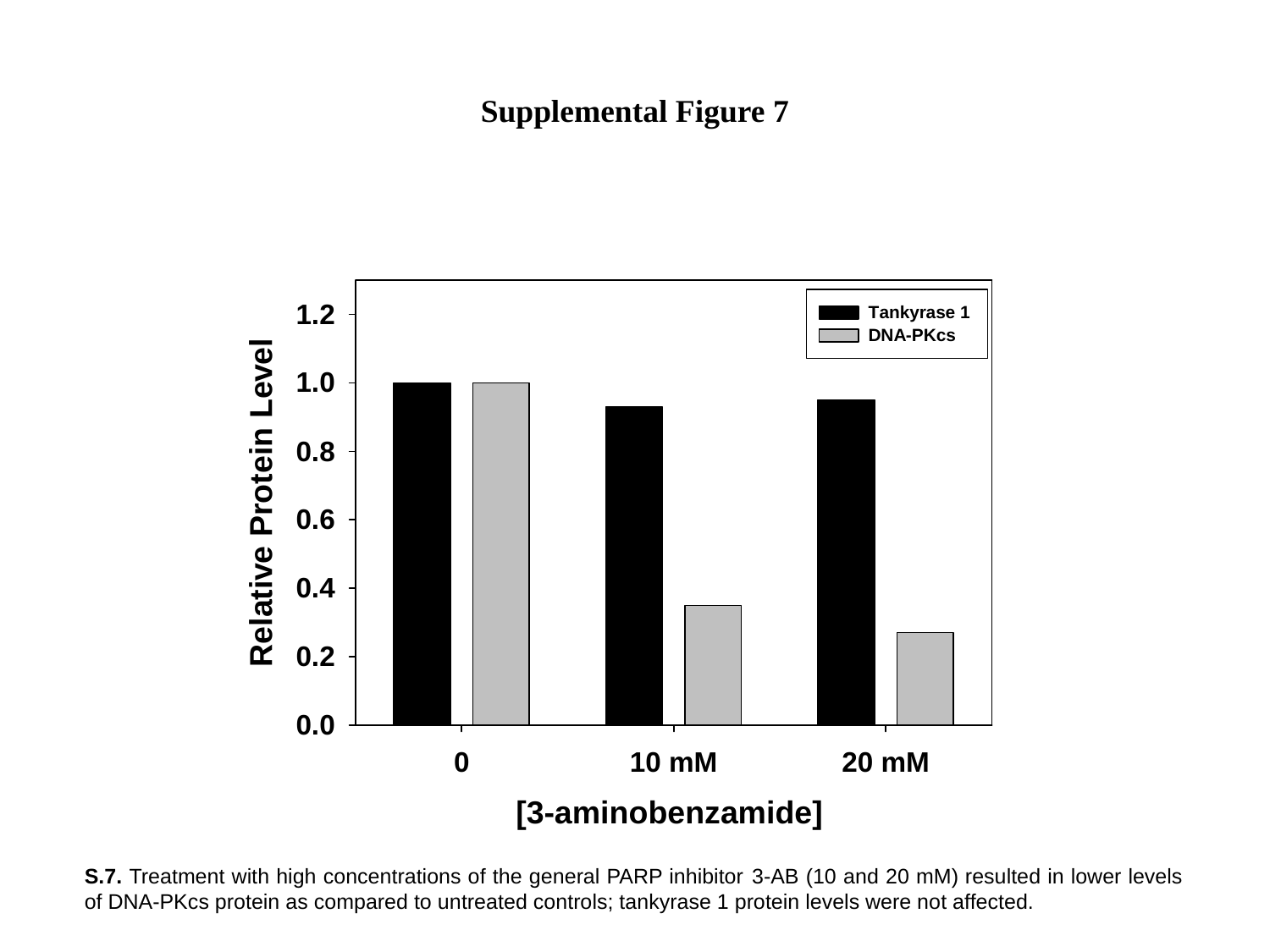

Supplemental Figure 7
S.7. Treatment with high concentrations of the general PARP inhibitor 3-AB (10 and 20 mM) resulted in lower levels of DNA-PKcs protein as compared to untreated controls; tankyrase 1 protein levels were not affected.

## Slide 10
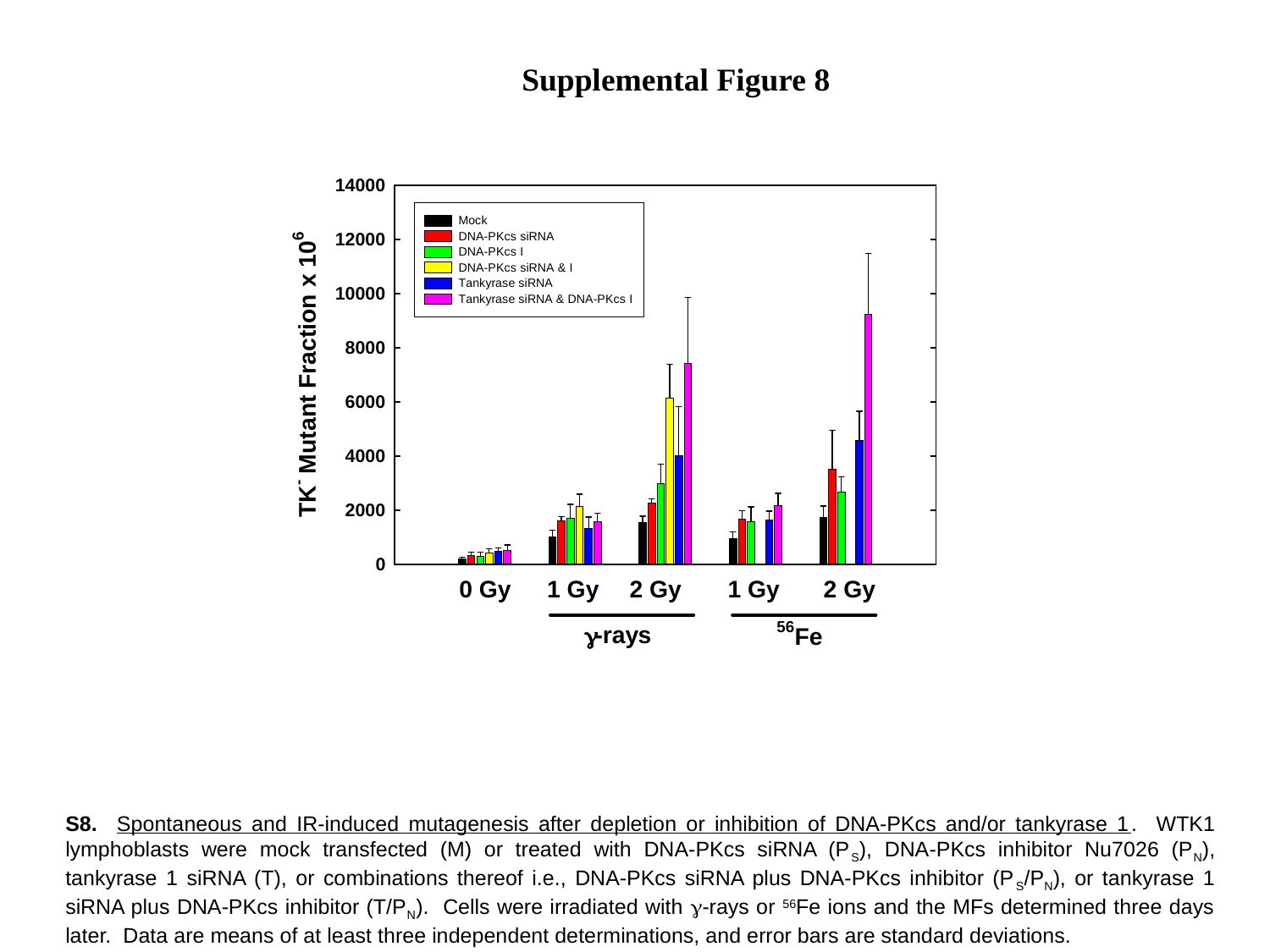

Supplemental Figure 8
S8. Spontaneous and IR-induced mutagenesis after depletion or inhibition of DNA-PKcs and/or tankyrase 1. WTK1 lymphoblasts were mock transfected (M) or treated with DNA-PKcs siRNA (PS), DNA-PKcs inhibitor Nu7026 (PN), tankyrase 1 siRNA (T), or combinations thereof i.e., DNA-PKcs siRNA plus DNA-PKcs inhibitor (PS/PN), or tankyrase 1 siRNA plus DNA-PKcs inhibitor (T/PN). Cells were irradiated with γ-rays or 56Fe ions and the MFs determined three days later. Data are means of at least three independent determinations, and error bars are standard deviations.
